# Molecular Basis and Regulation of OTULIN-LUBAC Interaction

**DOI:** 10.1016/j.molcel.2014.03.018

**Published:** 2014-05-08

**Authors:** Paul R. Elliott, Sofie V. Nielsen, Paola Marco-Casanova, Berthe Katrine Fiil, Kirstin Keusekotten, Niels Mailand, Stefan M.V. Freund, Mads Gyrd-Hansen, David Komander

**Affiliations:** 1 Medical Research Council Laboratory of Molecular Biology, Francis Crick Avenue, Cambridge, CB2 0QH, UK; 2 Department of Disease Biology, Novo Nordisk Foundation Center for Protein Research, University of Copenhagen, 2200 Copenhagen, Denmark

## Abstract

The linear ubiquitin (Ub) chain assembly complex (LUBAC) generates Met1-linked “linear” Ub chains that regulate the activation of the nuclear factor κB (NFκB) transcription factor and other processes. We recently discovered OTULIN as a deubiquitinase that specifically cleaves Met1-linked polyUb. Now, we show that OTULIN binds via a conserved PUB-interacting motif (PIM) to the PUB domain of the LUBAC component HOIP. Crystal structures and nuclear magnetic resonance experiments reveal the molecular basis for the high-affinity interaction and explain why OTULIN binds the HOIP PUB domain specifically. Analysis of LUBAC-induced NFκB signaling suggests that OTULIN needs to be present on LUBAC in order to restrict Met1-polyUb signaling. Moreover, LUBAC-OTULIN complex formation is regulated by OTULIN phosphorylation in the PIM. Phosphorylation of OTULIN prevents HOIP binding, whereas unphosphorylated OTULIN is part of the endogenous LUBAC complex. Our work exemplifies how coordination of ubiquitin assembly and disassembly activities in protein complexes regulates individual Ub linkage types.

## Highlights

• OTULIN binds the HOIP PUB domain via a conserved N-terminal PUB-interacting motif
• Structural studies reveal specificity determinants for the binary interaction
• Loss of HOIP-OTULIN interaction causes deregulated accumulation of Met1-polyUb
• OTULIN binding to LUBAC is regulated by phosphorylation

## Graphical Abstract

**Figure:**
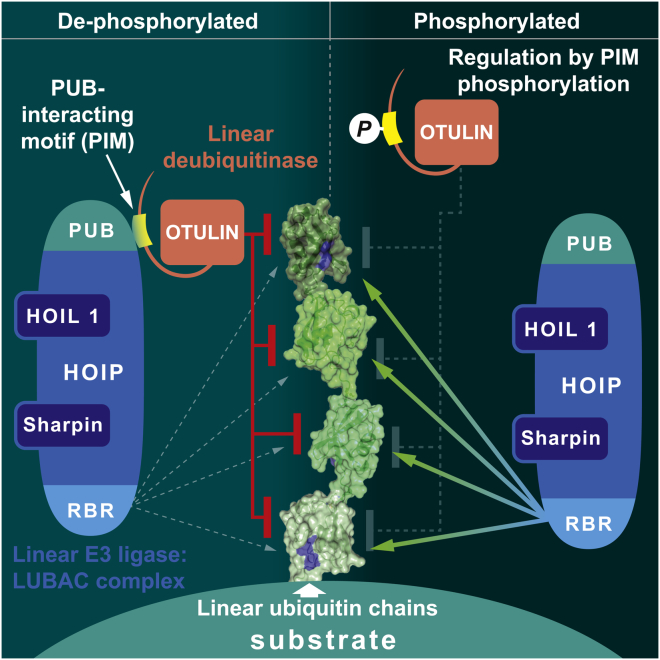

## Teaser

Elliott et al. show how the linear polyubiquitin-specific deubiquitinase OTULIN binds the linear ubiquitin chain assembly complex (LUBAC). The highly specific nanomolar interaction is mediated by the PUB domain of HOIP and an internal PUB-interacting motif (PIM) in OTULIN. Phosphorylation of the Tyr in the PIM regulates endogenous complex formation.

## Introduction

Protein ubiquitination is a versatile posttranslational modification in which Lys residues of substrates are modified with the small protein ubiquitin (Ub). Ub can be ubiquitinated itself, giving rise to polyUb chains. PolyUb chains regulate a wide variety of cellular processes ranging from protein degradation to activation of cellular signaling pathways (Hershko and Ciechanover, 1998; Komander and Rape, 2012). Because Ub itself has eight modification sites, a great variety of homotypic and heterotypic chains exist. It is becoming increasingly clear that different polyUb chains encode distinct signals and are independently and specifically assembled, recognized, and disassembled (Behrends and Harper, 2011; Kulathu and Komander, 2012). The most well-studied polyUb signals are Lys48-linked chains that mediate proteasomal degradation (Hershko and Ciechanover, 1998) and Lys63-linked chains that have various nondegradative roles in nuclear factor κB (NFκB) and other signaling pathways and in the DNA damage response (Chen and Sun, 2009).

Met1-linked or linear chains constitute a further important chain type in NFκB signaling (Tokunaga and Iwai, 2012; Walczak et al., 2012). Work by Kirisako et al. (2006) identified the linear Ub chain assembly complex (LUBAC), which consists of the chain-assembling E3 ligase HOIP as well as HOIL-1 and SHARPIN (Walczak et al., 2012). Importantly, deletion of the LUBAC component SHARPIN in mice (Gerlach et al., 2011; Ikeda et al., 2011; Tokunaga et al., 2011), or mutation of HOIL-1 in humans (Boisson et al., 2012), lead to hyperinflammatory phenotypes, indicating key roles of LUBAC and linear Ub chains in the response to infection and inflammation.

The remarkable specificity of HOIP for assembling Met1-linked chains resides in its RBR E3 ligase domain and a conserved C-terminal extension (Smit et al., 2012; Stieglitz et al., 2012b) and is now understood in molecular detail (Stieglitz et al., 2013). HOIP also comprises several NPL4 zinc finger (NZF) Ub binding domains (UBDs) that target it to ubiquitinated proteins (Haas et al., 2009), a Ub-associated (UBA) domain that mediates interactions with HOIL-1 (Yagi et al., 2012), and N-terminal PUB (peptide:N-glycanase/UBA- or UBX-containing proteins) and B box domains of unknown functions. PUB domains interact with the C terminus of the AAA+ ATPase p97 (also known as VCP, or cdc48 in yeast), which itself regulates a myriad of cellular signaling pathways, often in conjunction with the Ub system (Meyer et al., 2012).

Until recently, it was unclear how Met1-linked polyUb chains are hydrolyzed, given that many deubiquitinating enzymes (DUBs) are isopeptide specific and unable to hydrolyze Gly76-Met1 peptide bonds in Met1-linked chains (Komander et al., 2009; Mevissen et al., 2013). The majority of USP domain DUBs hydrolyze Met1 linkages with significantly lower activity in comparison to Lys linkages (Faesen et al., 2011).

Two reports recently identified FAM105B/OTULIN as an OTU domain DUB with high activity and unique specificity for Met1-linked polyUb (Keusekotten et al., 2013; Rivkin et al., 2013). OTULIN and LUBAC have coevolved in higher eukaryotes, and OTULIN antagonizes processes involving LUBAC, including tumor necrosis factor α (TNFα), poly(I:C), and NOD2 signaling (Fiil et al., 2013; Keusekotten et al., 2013). OTULIN was also implicated in angiogenesis and may affect Wnt signaling (Rivkin et al., 2013).

Knockdown of OTULIN or overexpression of a catalytically inactive mutant results in increased ubiquitination of proteins with Met1 linkages and leads to the ubiquitination of LUBAC itself. This suggests that OTULIN protects LUBAC from autoubiquitination (Fiil et al., 2013; Keusekotten et al., 2013). Moreover, immunoprecipitation of SHARPIN copurified HOIP, HOIL-1 and OTULIN (Keusekotten et al., 2013), and OTULIN interacted with HOIP in proteomic experiments (Fu et al., 2014; Rivkin et al., 2013), indicating that OTULIN may associate with HOIP and/or LUBAC.

Here, we show that OTULIN interacts directly with the N-terminal PUB domain of HOIP via a conserved PUB-interacting motif (PIM) in OTULIN. The OTULIN PIM is necessary and sufficient to establish a high-affinity interaction with HOIP, which is >40-fold higher in affinity than a HOIP-p97 interaction. Structural studies explain this high affinity and the OTULIN-HOIP specificity. Point mutants on either side of the interface disrupt the interaction in vitro and in cells. Loss of the HOIP-OTULIN interaction disables OTULIN-dependent regulation of HOIP ubiquitination and OTULIN’s capacity to efficiently shutdown LUBAC-induced NFκB activation, suggesting that OTULIN needs to be present on LUBAC to restrict Met1-polyUb signaling. Furthermore, complex formation is regulated by PIM phosphorylation.

## Results

### Identification of a HOIP-OTULIN Interaction

**Figure 1:**
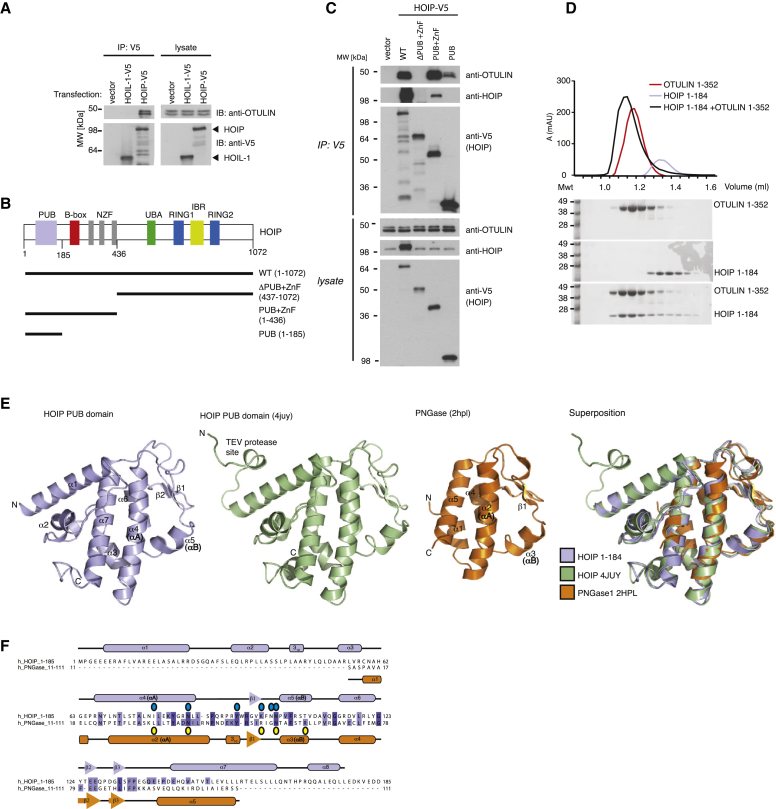
OTULIN Binds the HOIP PUB Domain (A) Epitope-tagged HOIP or HOIL-1 were transfected into HEK293T cells, and interaction with endogenous OTULIN was determined by immunoprecipitation followed by western blot analysis. OTULIN interacts with HOIP but not HOIL-1 under these conditions. (B) Domain representation of HOIP. A bar graph below indicates constructs used for domain mapping. (C) Domains of epitope-tagged HOIP were transfected into U2OS and NOD2 cells and probed for endogenous OTULIN following the coimmunoprecipitation described in (A). (D) Analytical size-exclusion chromatography profile of HOIP 1-184 (blue), full-length OTULIN (red), and 1:1.2 OTULIN:HOIP complex (black). Coomassie-stained SDS-PAGE gels below show protein-containing fractions. (E) Left, extended HOIP PUB domain structure (blue). Middle left, HOIP PUB domain structure determined by the SGC (green, PDB ID 4JUY). Middle right, structure of PNGase PUB domain (orange, PDB ID 2HPL) (Zhao et al., 2007). Right, superposition. The SGC-determined HOIP structure includes an additional TEV protease cleavage site at the N terminus (see also Figure S5B). (F) Structure-based sequence alignment of HOIP and PNGase PUB domains. HOIP contains two additional N-terminal helices and an additional C-terminal helix not found in PNGase. Open circles represent residues in HOIP (blue), and PNGase (yellow) that interact with the OTULIN/p97 PIMs, respectively.

Previous studies of OTULIN had suggested an interaction between OTULIN and LUBAC; however, although Rivkin et al. (2013) speculated that OTULIN forms a subcomplex with HOIP alone, we showed that SHARPIN immunoprecipitated OTULIN, HOIL-1, and HOIP (Keusekotten et al., 2013). Indeed, immunoprecipitation of overexpressed HOIP, but not HOIL-1, copurified endogenous OTULIN (Figure 1A). HOIP truncations were used to map the region of HOIP that interacts with OTULIN. This indicated that the N-terminal 185 amino acids (aa) spanning the PUB domain of HOIP were sufficient to coimmunoprecipitate endogenous OTULIN (Figures 1B and 1C). OTULIN interaction was increased in longer constructs (aa 1–436, also including B box and NZF domains); however, this longer construct also interacted with endogenous HOIP, suggesting that it harbors the oligomerization module of HOIP and that oligomerization of HOIP most likely enhances OTULIN binding (Figure 1C). Next, the HOIP-OTULIN interaction was verified in vitro. Constructs spanning the annotated PUB domain (aa 67–158) were insoluble, but including the conserved HOIP N terminus resulted in a stable fragment of HOIP (Figure S1A available online). This extended PUB domain construct (aa 1–184) is able to bind full-length OTULIN in analytical size-exclusion chromatography studies (Figure 1D).

### Structure of the HOIP PUB Domain

To understand structural features of the extended HOIP PUB domain, we crystallized and determined its structure to 3.0 Å resolution by molecular replacement with the use of coordinates deposited by the Structural Genomics Consortium (SGC; Protein Data Bank [PDB] ID 4JUY Figures 1E and S1B and Table 1). Our structure contains 13 molecules within the asymmetric unit that superimpose with a low root-mean-square deviation (rmsd; 0.9–1.2 Å; Figure S1C). As anticipated, residues 59–158 of HOIP form a PUB domain resembling that of PNGase, the only other PUB domain structurally characterized to date (Allen et al., 2006; Zhao et al., 2007). HOIP and PNGase superimpose with an rmsd of 7.2 Å for residues 59–158 of HOIP (Figure 1E), and most secondary structure elements are conserved (Figure 1F). In addition, the HOIP PUB domain contains two N-terminal helices and one C-terminal helix that contribute to the hydrophobic core of the PUB domain, revealing why shorter constructs were insoluble (Figure 1F). Hydrophobic residues within the N-terminal extension are conserved among most HOIP orthologs, suggesting that the extended fold is conserved (Figure S1A). A similar extension is not present in the N-terminal PUB domain of PNGase (Allen et al., 2006; Zhao et al., 2007) or in the only other protein in which a PUB domain has been annotated, UBXD1 (Kern et al., 2009) (Figure S1D). Consistently, a minimal UBXD1 PUB domain (aa 150–264) is soluble and functional (see below).

### Functional Surfaces in the HOIP PUB Domain

**Figure 2:**
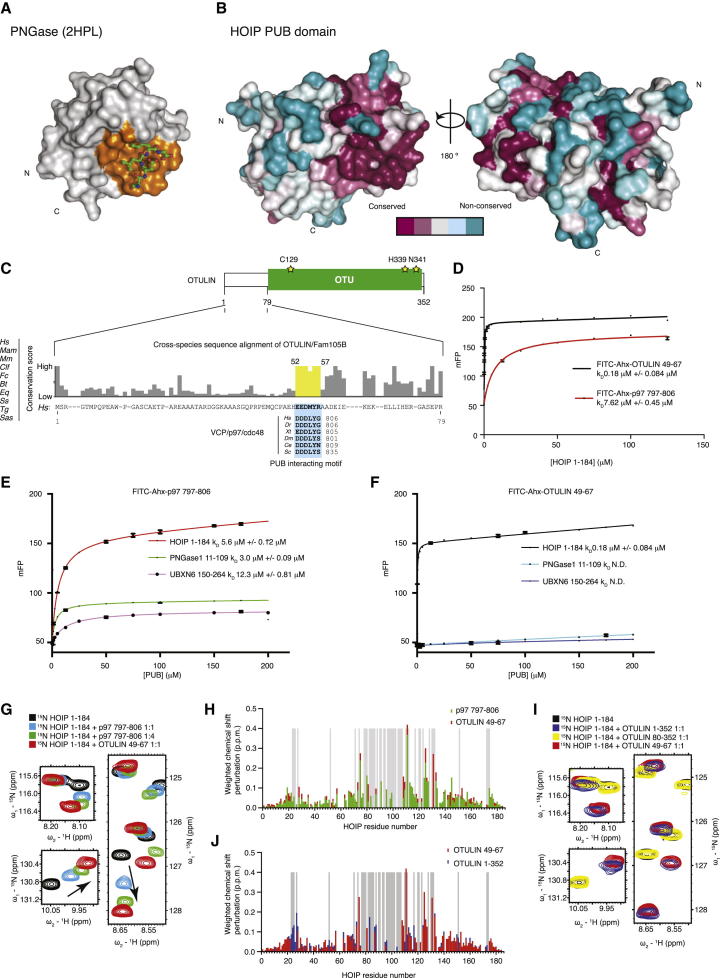
A PUB-Domain-Interacting Motif in OTULIN (A) Structure of PNGase bound to the p97 PIM peptide (PDB ID 2HPL) (Zhao et al., 2007) reveals the position of the PIM pocket. (B) Surface conservation analysis of the HOIP PUB domain colored according to the sequence alignment in Figure S1A. The PIM pocket is highly conserved, whereas other regions, including the surface generated by the N-terminal PUB domain extension, are not conserved. (C) Primary sequence alignment of the HOIP binding region in OTULIN (Figure S3A) (Rivkin et al., 2013). Alignment shows that the patch with highest evolutionary conservation resembles the p97 PIM. (D) Affinity measurements using HOIP PUB domain against FITC-Ahx-labeled p97 PIM peptide (aa 797–806) and OTULIN PIM peptide (aa 49–67). Experiments were performed in triplicate, and errors represent SD from the mean. (E) Binding of PUB domains from HOIP (aa 1–184, red), PNGase (aa 11–109, green), and UBXD1 (aa 150–264, magenta) to a fluorescent p97 PIM peptide (aa 797–806) as in (D). *K*_*D*_ values are indicated, and errors represent SD from the mean from triplicate experiments. (F) Binding of PUB domains as in (E) to the OTULIN PIM peptide. (G) ^15^N-transverse relaxation optimized spectroscopy (TROSY) spectra of HOIP alone (black), HOIP bound to OTULIN PIM peptide at a 1:1 molar ratio (red), and HOIP bound to p97 PIM peptide at a 1:1 (blue) and 1:4 ratio (green). Selected perturbed resonances are shown. For the full spectra, see Figure S4A. (H) Chemical shift map by HOIP residue number for perturbation by p97 and OTULIN PIM peptides. (I) ^15^N-TROSY spectra of HOIP alone (black), HOIP bound to OTULIN PIM peptide (red), HOIP bound to full-length OTULIN (blue), and HOIP with OTULIN catalytic domain (aa 80–352, yellow), all at a 1:1 molar ratio. The same resonances as in (G) are shown. For the full spectra, see Figures S4B, 4C, and 3. (J) Difference map of chemical shifts between HOIP bound to PIM peptide or full-length OTULIN derived from respective spectra in (I).

The PNGase PUB domain was shown to have two functional surfaces. The first one is the PIM pocket derived from a PNGase crystal structure in complex with a five-residue DDLYG PIM peptide corresponding to the p97 C terminus (Zhao et al., 2007). In this interaction, two key residues in the PIM peptide (Leu804 and Tyr805) form mainly hydrophobic interactions with a hydrophobic pocket, the PIM pocket (Zhao et al., 2007) (Figures 1F and 2A, see below). A second functional surface of the PNGase PUB domain is a binding site for Ub or the Ub-like domain of human Rad23 located on the opposite face of the PIM pocket (Kamiya et al., 2012).

To understand whether these functional surfaces were conserved in HOIP, we analyzed surface conservation of its PUB domain (Figures 2B and S1A). Most surface residues in HOIP, including those potentially involved in Ub interaction, are not conserved. Consistently, we were unable to detect binding of the HOIP PUB domain to Ub or Met1-linked diUb by nuclear magnetic resonance (NMR) analysis (Figure S2).

In contrast, the residues forming a putative PIM pocket are highly conserved in HOIP. The HOIP PIM pocket is formed by hydrophobic residues located on helices α4 (equivalent to helix α2 in PNGase, hereafter named αA) and α5 (equivalent to helix α3 in PNGase, hereafter named αB) and on the β1 strand (compare Figures 2B and 1E). This suggested that the PIM pocket in HOIP is most likely important for OTULIN binding and that OTULIN might contain a PIM.

### Identification of a PIM in OTULIN

Using analytical size-exclusion chromatography analysis, we mapped the HOIP interaction site of OTULIN to its N-terminal 80 aa, which was in agreement with previous data (Rivkin et al., 2013) (Figure S3A). Closer inspection of this region revealed low overall conservation, with the exception of a short invariant EEDMYR motif spanning residues 52–57 that resembled the p97 PIM (Figure 2C). We used a fluorescence polarization assay to test whether FITC-labeled OTULIN (aa 49–67) or p97 (aa 797–806) (Zhao et al., 2007) peptides were able to bind the HOIP PUB domain. The p97 peptide bound to the HOIP PUB domain with 7.6 μM affinity, which is similar to other PUB-p97 interactions (Figure 2D, see below). Importantly, the OTULIN peptide bound HOIP with 180 nM affinity, a >40-fold increase in comparison to p97 (Figure 2D).

The realization that OTULIN contains a PIM immediately raised the intriguing possibility that OTULIN might interact with other PUB-domain-containing proteins. Hence, the binding of PIM peptides of OTULIN and p97 to the PUB domains of HOIP, PNGase, or UBXD1 was compared. All three domains bound fluorescently labeled p97 PIM peptide with similar affinity (3 μM for PNGase, 6 μM for HOIP, and 12 μM for UBXD1), which was in accordance with published isothermal titration calorimetry data (3 μM for PNGase; Figure 2E) (Zhao et al., 2007). Interestingly, the OTULIN PIM bound to HOIP, but not to UBXD1 or PNGase, PUB domains (Figure 2F).

### Characterization of the HOIP-OTULIN Complex by NMR

We used NMR to further understand the molecular basis of the OTULIN-HOIP interaction. A ^15^N-labeled HOIP PUB domain construct (aa 1–184) was analyzed by BEST-TROSY (Solyom et al., 2013), revealing well-dispersed peaks (Figure 2G). Triple-resonance experiments with ^13^C- and ^15^N-labeled HOIP PUB domain protein allowed the assignment of 167 out of 186 amino acids.

Significant chemical shift perturbations (CSPs) were observed when unlabeled PIM peptides derived from OTULIN or p97 were added to labeled HOIP PUB domain (Figures 2G and 2H). Both peptides resulted in qualitatively identical CSPs (Figure 2H), suggesting similar binding modes. However, although the p97 peptide displayed CSPs indicative of fast-exchange behavior on the NMR time scale, the OTULIN peptide showed CSPs and loss of a large number of resonances, a feature common to slow exchange (Figure 2G, see also Figure S4A). This is consistent with a >40-fold higher affinity of the OTULIN peptide as observed by fluorescence polarization, and it most likely reflects a higher dynamic equilibrium for the p97 PIM peptide in comparison to a more stable interaction with the OTULIN PIM. A comparison of ^13^C-HSQC spectra, which monitor aliphatic side chain resonances, showed that only a small subset of peaks were perturbed. This indicated that peptide binding did not result in large-scale conformational changes in the HOIP PUB domain (Figure S3B).

Next, we tested whether the extended HOIP PUB domain interacted exclusively via the PIM or whether it formed additional interactions with the OTULIN OTU domain. For this, ^15^N-labeled HOIP PUB domain was mixed with full-length OTULIN (aa 1–352), OTULIN ovarian tumor (OTU) domain (aa 80–352), or the OTULIN PIM peptide (aa 49–67, see above). A comparison of the resulting spectra confirmed that the OTU domain did not interact with the HOIP PUB domain (Figures 2I, see Figures S4B and S4C for the full spectra). Importantly, the pattern of HOIP CSPs was identical upon the addition of either OTULIN PIM peptide or full-length OTULIN (Figures 2I and 2J). Moreover, despite forming a ∼60 kDa complex, the spectra of the HOIP PUB domain were unaffected by line broadening, indicating that the PIM in OTULIN is quasi-independent from the OTU domain and displays the dynamic behavior of a small protein (Figures 2H and S4C). This revealed that the PIM is the sole binding site between OTULIN and the HOIP PUB domain.

### Structure of the HOIP PUB Domain in Complex with the OTULIN PIM

**Figure 3:**
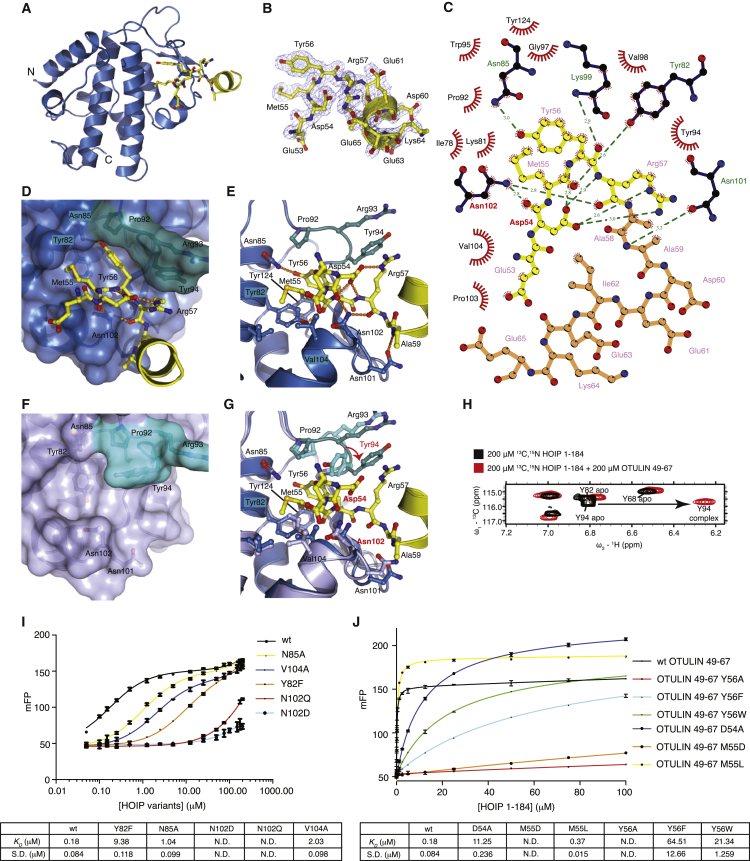
Structure of HOIP Bound to OTULIN Peptide (A) Structure of HOIP PUB domain (aa 5–180; blue) bound to the OTULIN PIM peptide (yellow). The peptide is in ball-and-stick representation with blue nitrogen and red oxygen atoms. (B) A weighted 2|Fo|-|Fc| map contoured at 1 σ covering the OTULIN PIM peptide colored as in (A). (C) LIGPLOT representation of the HOIP-OTULIN interaction. Residues in the PIM (aa 53–57) are shown in yellow, and the C-terminal extension of the PIM is shown in orange. Hydrogen bonds are shown by green dashes, and van der Waals contacts are shown as red fans. (D) PIM pocket shown in surface representation in the HOIP-OTULIN complex colored as in (A). Residues 92–94, including the mobile Tyr94, are colored green. (E) Close-up view of the OTULIN PIM peptide in the HOIP PIM pocket, colored as in (A), showing hydrogen bonds as orange dotted lines. (F) PIM pocket shown in surface representation in apo HOIP, in which Tyr94 (green) partly occludes the PIM pocket. (G) Superposition of apo and PIM-peptide-bound HOIP highlighting the conformational change in Tyr94 side chain. (H) A conformational change of the Tyr94 side chain is resolved in the aromatic region of ^13^C-HSQC spectra, with HOIP alone (black) and the HOIP OTULIN PIM complex spectrum shown in red. The shifting resonance indicated by an arrow corresponds to the Cε of Tyr94. Only the Cε region of the aromatic ^13^C-HSQC is shown. (I) Fluorescent polarization assay of wild-type OTULIN PIM peptide binding to purified HOIP (aa 1–184) PIM pocket mutants. Binding parameters are listed below. Experiments were performed in triplicate, and errors represent SD from the mean. (J) Binding of HOIP PUB domain (aa 1–184) to OTULIN peptides (aa 49–67) with the indicated point mutations in the PIM performed as in (I). Binding parameters are listed below.

Having established the minimal requirements for the HOIP-OTULIN interaction, we set out to crystallize the complex. We determined the structure of a slightly truncated HOIP PUB domain construct (aa 5–180) bound to the OTULIN PIM peptide (aa 49–67) to 2.0 Å resolution (Figure 3A, Table 1). The two molecules in the asymmetric unit were highly similar to the apo structures (rmsd ∼1.5 Å; Figure S5A), which was consistent with NMR analysis (Figure S3B). Both HOIP molecules showed similarly well-defined electron density for residues 53–65 of the PIM peptide (Figure 3B). In analogy to the p97-PNGase interaction, only PIM residues 54–58 interact with HOIP. The PIM peptide forms a 90° kink, in which Met55 and Tyr56 form a bulge and mediate key hydrophobic interactions. Residues 49–52 and 66–67 are disordered in the crystal structure, and residues 53 and 59–65 protrude from the PUB domain without forming contacts.

As expected, the OTULIN PIM peptide binds to the conserved PIM pocket in the HOIP PUB domain (Figures 2B and 3). The key PIM residue Tyr56 is buried in a pocket formed by Tyr82 (αA), Tyr124, and Pro92 and formed a hydrogen bond with Asn85 from HOIP. The second hydrophobic PIM residue, Met55, is bound in a shallower groove between HOIP PUB domain residues Tyr82, Ile78 (αA), and Val104 (αB; Figure 3D). In addition to these hydrophobic contacts, HOIP also forms a total of six hydrogen bonds with the backbone of the PIM peptide (Figures 3C and 3E).

Of special interest are Asp54 in the OTULIN PIM peptide and Asn102 in HOIP, given that these residues induce the 90° kink in the PIM peptide. Asp54 in the peptide binds in *cis* to the backbone amides of OTULIN Tyr56 and Arg57 and to the δ-guanidyl group of Arg57. More importantly, Asn102 in the PUB domain acts as the cornerstone around which the peptide is wrapped and interacts with the very same backbone of Asp54, Tyr56, and Arg57. Hence, Asp54 and Asn102 induce the required kinked conformation of the PIM peptide in *cis* and *trans*, respectively, indicating that Asn102 is a key residue in the interaction (Figure 3E).

Arg57 of the PIM peptide participates in a π-π stacking network with HOIP Tyr94, which is the only residue that undergoes a significant conformational change within the PIM pocket. In our apo structures, the side chain of Tyr94 is rotated to bind the HOIP Tyr pocket in *cis*, appearing to block access to the PIM pocket (Figure 3F). In the PIM peptide complex, a 90° rotation of the Tyr94 side chain displaces it from the PIM pocket (Figure 3G). Interestingly, Tyr94 is displaced from the PIM pocket in the apo structure determined by the SGC (PDB ID 4JUY). However, in this structure, residues from the tobacco etch virus (TEV) protease site constitute a pseudo-PIM and interact in *trans* with the PIM pocket of a neighboring molecule in the crystal lattice (Figure S5B).

We were able to independently verify the conformational change of Tyr94 upon PIM binding with the use of ^13^C-HSQC experiments that allow monitoring changes in aromatic residues. Tyr94 aromatic ring protons undergo significant CSPs upon PIM binding. This suggests conformational opening and closing of the PIM pocket in HOIP (Figure 3H).

### Probing the HOIP-OTULIN Interaction

The observed binding modes of the OTULIN PIM peptide with the HOIP PUB domain were validated by mutational analysis. Mutations that affect the size and shape of the hydrophobic PIM pocket (Y82F, V104A, and N85A) reduced binding affinities 10- to 50-fold (Figure 3I). Importantly, even conservative mutation of the aforementioned cornerstone residue Asn102 to Asp (N102D) or Gln (N102Q) abolished HOIP binding to OTULIN (Figure 3I).

To test mutations in OTULIN, we synthesized fluorescently labeled OTULIN peptides with point mutations in Tyr56 (Y56A, Y56F, and Y56W), Met55 (M55D), and Asp54 (D54A). As anticipated, Y56A and M55D mutations abrogated binding, whereas Y56F or Y56W mutation greatly reduced binding (>400- and 100-fold, respectively). Destabilization of the Asp54-induced conformation of the PIM peptide resulted in a 60-fold reduction of HOIP binding (Figure 3J), indicating that stabilizing the kink in the PIM peptide is crucial for PUB interaction.

### Understanding OTULIN-HOIP Specificity

Although the structural data revealed the molecular basis for HOIP-OTULIN interaction, a number of questions regarding the observed specificity of the interaction remained. In particular, HOIP bound p97 with >40-fold reduced affinity in comparison to OTULIN, and the reason for this difference must reside in the distinct PIMs of the two proteins. Second, although p97 was promiscuous, OTULIN was unable to bind other PUB domains, indicating key differences in the involved PUB domains.

#### Understanding HOIP Specificity for OTULIN

**Figure 4:**
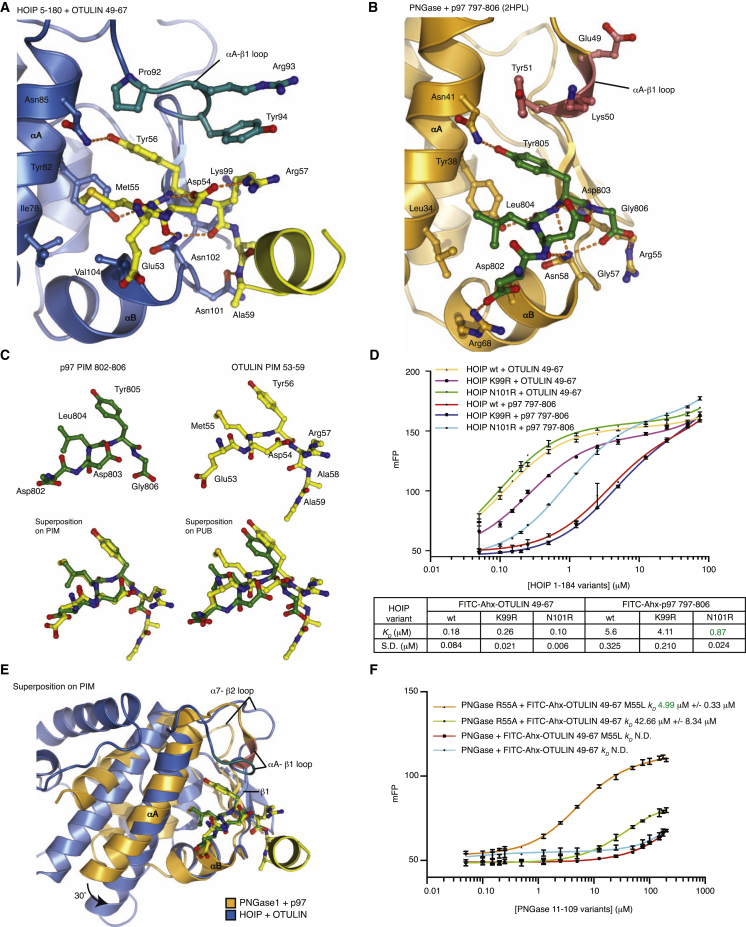
Specificity of the HOIP-OTULIN Interaction (A) Close-up view of the HOIP PUB domain (blue) bound to OTULIN PIM (yellow) as in Figure 3E. Interacting residues are shown in ball-and-stick representation and labeled. Hydrogen bonds are indicated as orange dotted lines. (B) Same view as in (A) for the PNGase-p97 complex (PDB ID 2HPL) (Zhao et al., 2007). Residues 49–51 that differ structurally from HOIP are colored red. (C) PIM peptides from p97 (green) and OTULIN (yellow) can be perfectly superimposed (bottom left) but do not align once PUB domains are superposed because of deeper binding of the OTULIN PIM in the HOIP PIM pocket. (D) Fluorescence polarization assays of HOIP N101R/K99R mutants with FITC-Ahx-labeled p97 (797–806) or OTULIN (49–67) PIMs as described in Figure 2D. Binding parameters are listed below. Experiments were performed in triplicate, and errors represent SD from the mean. (E) Superposition on the PIM of PNGase-p97 (orange and green) and HOIP-OTULIN (blue and yellow) shows perfect alignment of the Asn cornerstone residue but also the misalignment of PUB domain core helices, indicating different binding modes. (F) Fluorescence polarization assays of PNGase and FITC-Ahx-labeled OTULIN (49–67) with point mutations in the PUB domain and the PIM peptide that promote binding of OTULIN PIM to PNGase. Experiments were performed in triplicate, and errors represent SD from the mean.

To understand these specificity considerations, we compared the binding modes of OTULIN-HOIP to those of p97-PNGase (Figures 4A and 4B). The key differences in the OTULIN PIM peptide are the C-terminal extension not present in the C-terminal p97 peptide and the exchange of Leu-Tyr-Gly in p97 for Met-Tyr-Arg in OTULIN. Apart from this, the PIM peptides can be superimposed well (Figure 4C).

A fluorescently labeled OTULIN peptide in which Met55 was exchanged to Leu (as in p97) bound HOIP with near-identical affinity (370 nM), showing that the small change in the first hydrophobic residue did not account for the difference (Figure 3J). Next, we speculated that HOIP did not form similar interactions with the p97 C terminus. In PNGase, this group forms two hydrogen bonds with the PUB domain residue Arg55 (Figure 4B). In HOIP, the equivalent position is Lys99, the side chain of which does not interact with the OTULIN PIM (Figure 4A). K99R mutation had similar marginal effects on OTULIN or p97 interaction (Figure 4D). HOIP uses Asn101 to bind to Ala59 of the OTULIN PIM (Figure 4A), which has no equivalent in the p97 PIM (Figure 4B), and Asn101 would be too far to contact the p97 C terminus. Importantly, mutation of HOIP Asn101 to Arg improved p97 binding 9-fold (from 7.6–0.9 μM; Figure 4D), suggesting that the introduced Arg101 contacts the p97 C terminus and now contributes to the interaction. Interestingly, the N101R mutation does not significantly affect OTULIN interaction (180 versus 100 nM; Figure 4D), suggesting that HOIP has selectively weakened p97 interaction in order to gain specificity for OTULIN.

Despite the high-affinity, and seemingly more stable, interaction between HOIP and OTULIN, the interaction between HOIP and p97 was still significant and similar to other PUB-p97 interactions (Figure 2E). To test whether p97 can still bind HOIP in the presence of OTULIN, we measured its ability to compete for the PIM pocket in a fluorescence polarization competition assay. Interestingly, the p97 PIM peptide competed poorly with the OTULIN PIM for the HOIP binding site (*K*_*i*_ of 37 μM; Figure S6A). This strengthens the observation that the HOIP-OTULIN interaction is significantly more stable than a HOIP-p97 interaction.

#### Understanding OTULIN Specificity for HOIP

Differences in the PIM pocket of HOIP and PNGase explain the observed specificity of OTULIN for the HOIP PUB domain. Superposition of the PIM peptides in both complexes aligns the αB helices containing the crucial cornerstone Asn residues and the β1 strands. However, the remaining core helices including αA display a ∼30° rotation, leading to a different overall disposition of hydrophobic residues (Figure 4E). This suggests the presence of a hinge between the helical core (including αA) of the PUB domain and the αB-β1 subdomain. Indeed, the loops between αA and β1 are well ordered, conserved, and conformationally identical in all structures of the respective PUB domains but structurally highly divergent in HOIP and PNGase (Figure 4E). The HOIP αA-β1 loop contains Tyr94 that undergoes a conformational change upon PIM binding (see above). In contrast, the equivalent Tyr51 in PNGase provides a seemingly solid sidewall to the PIM pocket and is conformationally rigid. This difference in Tyr positioning and flexibility shapes the PIM pocket, which is deeper in HOIP than it is in PNGase. Consistently, superposition of the PUB domains reveals that the OTULIN PIM has moved by 1.5 Å deeper into the HOIP PIM pocket, most likely explaining the observed high affinity for the OTULIN-HOIP interaction (Figures 4C and S6B).

Moreover, this difference in size and shape of the PIM pocket explains why PNGase cannot bind OTULIN. Although superposition of the OTULIN PIM onto PNGase does not reveal significant clashes (Figure 4E), the larger Met in the OTULIN PIM (versus Leu in p97) may be too big for PNGase. However, a fluorescently labeled OTULIN PIM with M55L mutation that mimics the Leu-Tyr of the p97 sequence was still unable to bind PNGase (Figure 4F). Another key difference in the PUB domains is Arg55 in PNGase, which binds the C terminus and “closes” the PIM pocket, potentially disallowing the binding of C-terminally extended PIM peptides, as found in OTULIN. The equivalent Lys101 in HOIP points away from the PIM pocket (see above). Indeed, we started to detect an OTULIN-PNGase interaction when Arg55 was mutated to Ala (*K*_*D*_ 43 μM; Figure 4F). Importantly, when this PNGase mutant was tested with the OTULIN M55L PIM peptide, full binding was recovered (*K*_*D*_ 5 μM; Figure 4F). Hence, with point mutations in OTULIN to generate a more p97-like PIM and in PNGase to remove the requirement for a C-terminal PIM as in p97, we have engineered a μM binding interface in two proteins that did not interact previously. This confirms that the specificity of OTULIN for the HOIP PUB domains originates from a slightly larger PIM pocket in HOIP that allows binding of internal PIMs.

### Characterization of OTULIN-HOIP Interactions In Vivo

**Figure 5:**
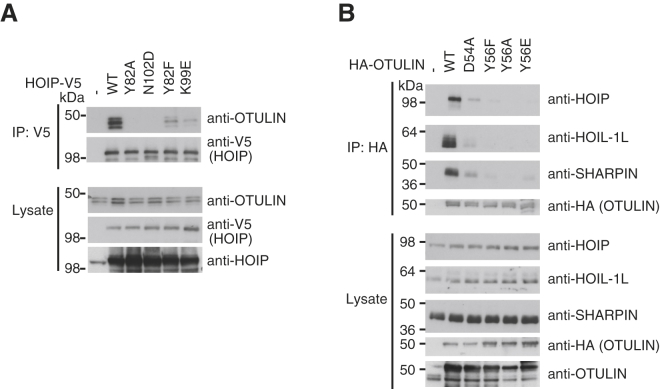
Verification of HOIP-OTULIN Interactions in Cells (A) Experiments performed as in Figure 1A with HOIP point mutations in the PIM pocket and testing the binding of endogenous OTULIN as detected by an OTULIN antibody. (B) HA-tagged OTULIN or OTULIN PIM mutants were expressed in HEK293T cells immunoprecipitated with anti-HA-Agarose resin, and LUBAC components HOIP, HOIL-1, and SHARPIN were detected by western blotting against endogenous components.

Having characterized the PUB-PIM interaction in vitro, we wondered whether it was responsible for HOIP-OTULIN interaction in cells. For this, we first overexpressed V5-tagged HOIP wild-type or HOIP with point mutations in the PUB binding site and then tested its ability to coimmunoprecipitate endogenous OTULIN. Although wild-type HOIP coprecipitated OTULIN, mutations Y82A and N102D abrogated OTULIN binding, and Y82F and K99E decreased binding (Figure 5A), which was consistent with the roles of these residues in PIM binding (see above).

For the reverse experiment, we overexpressed full-length OTULIN or OTULIN with point mutations in the PIM and monitored their interactions with endogenous LUBAC components. HA-tagged OTULIN coimmunoprecipitated all proteins from the endogenous LUBAC complex, whereas mutations of Tyr56 (Y56F, Y56A, and Y56E) abrogated binding. Residual binding was still observed with an OTULIN D54A mutant, which most likely only destabilizes the kink in the PIM peptide (see Figure 3J). This showed that the HOIP-OTULIN interaction in cells could be modulated by single point mutations on either side of the interface (Figure 5B).

### Functional Consequences of Modulating the HOIP-OTULIN Interface

**Figure 6:**
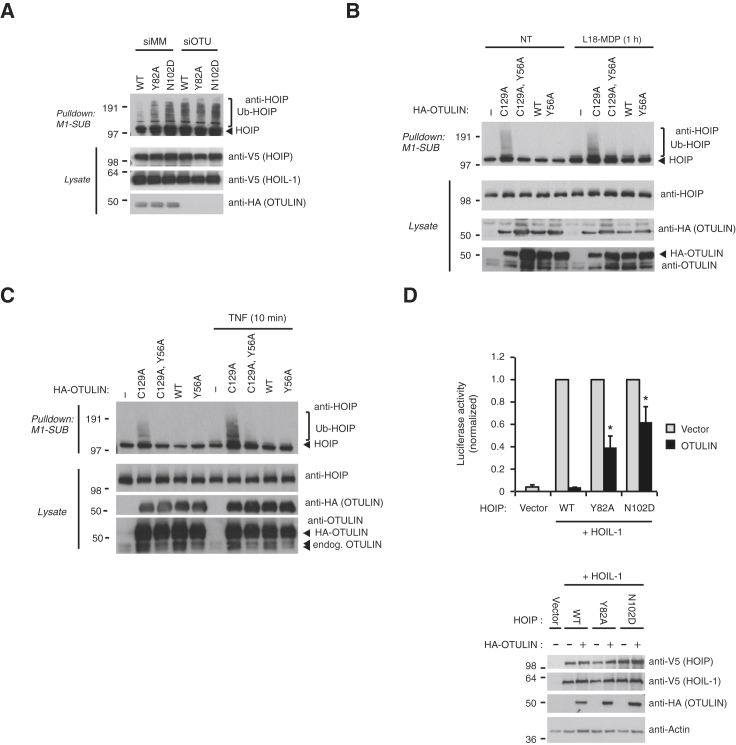
Functional Consequences of the OTULIN-LUBAC Interaction (A) Purification of endogenous Ub conjugates with Met1-specific Ub binding domain (Keusekotten et al., 2013) in lysates of HEK293T control and OTULIN-depleted cells transfected with HOIP variants and HOIL-1. Purified material and lysate was examined by immunoblotting. Mutation of the HOIP PIM pocket results in spontaneous accumulation of Met1-linked polyubiquitin on HOIP. (B and C) Purification of endogenous Ub conjugates with M1-SUB in U2OS and NOD2 cells transfected with the indicated OTULIN variants and treated with L18-MDP (B) or TNF (C). Purified material was analyzed as in (A). Mutation of the OTULIN PIM impairs stabilization of HOIP ubiquitination by catalytic inactive (C129A) OTULIN under basal conditions and after stimulation. (D) NFκB reporter activity in lysates of HEK293T cells transfected with HOIL-1, HOIP, or HOIP PIM pocket mutants and with or without the expression of OTULIN. OTULIN abrogated NFκB activity induced by wild-type LUBAC but was less effective in inhibiting activity induced by HOIP PIM pocket mutants.

So far, the cellular consequences of OTULIN-LUBAC interaction are unclear. We have previously shown that knockdown of OTULIN or overexpression of a catalytically inactive OTULIN C129A mutant (CA) lead to the autoubiquitination of HOIP with Met1-linked polyUb chains (Fiil et al., 2013; Keusekotten et al., 2013) (Figure 6A, compare lanes 1 and 4; Figure 6B, compare lanes 1 and 2). We wondered whether this depended on the formation of the OTULIN-HOIP complex or whether OTULIN would act in *trans* on the complex. When coexpressed with HOIL-1, HOIP PUB binding mutants autoubiquitinated in cells expressing endogenous OTULIN, and knockdown of OTULIN did not increase HOIP ubiquitination (Figure 6A). This observation suggests that, under basal conditions, the binding of OTULIN prevents HOIP autoubiquitination. Supporting this, ectopic expression of an inactive OTULIN with a mutation in the PIM (Y56A) did not lead to HOIP autoubiquitination, whereas inactive OTULIN with an intact PIM led to extensive HOIP ubiquitination (Figure 6B). Identical results were obtained when the activity of endogenous HOIP was induced by the NOD2 stimulus L18-MDP (Figure 6B) or treatment with TNF (Figure 6C). To investigate the functional importance of the HOIP-OTULIN interaction on NFκB signaling, we first coexpressed HOIP and HOIL-1 together with wild-type OTULIN or the PIM mutant Y56A. Although OTULIN Y56A was consistently slightly less potent in inhibiting NFκB activity in comparison to wild-type OTULIN, the assay revealed the difficulty in comparing different OTULIN variants functionally by overexpression (Figure S7), as reported previously (Rivkin et al., 2013).

Instead, we tested how mutations in the HOIP PUB binding site would affect the capacity of wild-type OTULIN to inhibit LUBAC-induced NFκB activity. Importantly, mutation of the cornerstone residue Asn102 to Asp (N102D) or a mutation that affect the hydrophobic PIM pocket (Y82A) reduced the ability of OTULIN to antagonize LUBAC-induced NFκB activity in comparison to wild-type HOIP (Figure 6D). This reveals that OTULIN has to be present on LUBAC in order to regulate NFκB signaling.

### Regulation of OTULIN-LUBAC Interaction by Phosphorylation

**Figure 7:**
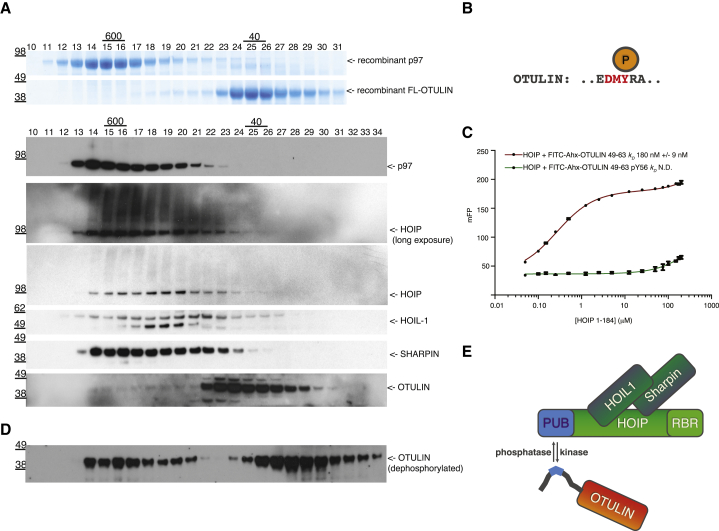
Regulation of OTULIN-LUBAC Complex Formation by Phosphorylation (A) Gel filtration analysis of purified bacterial p97 hexamers and full-length OTULIN visualized by Coomassie staining and HEK293ET cell lysates probed with indicated antibodies. (B) Schematic of the OTULIN PIM indicating phosphorylation at Tyr56 as identified in 22 independent mass spectrometry experiments in http://phosphosite.org/proteinAction.do?id=2470471. (C) Fluorescence polarization assays of HOIP PUB domains with wild-type and Tyr56-phosphorylated FITC-Ahx-labeled OTULIN (49–67). Experiments were performed in triplicate, and errors represent SD from the mean. (D) HEK293ET lysates were prepared in absence of phosphatase inhibitors and probed for the same components as in (A). Only the OTULIN blot is shown. (E) Schematic model of the LUBAC-OTULIN complex indicating its regulation by protein phosphorylation.

Next, we wondered whether OTULIN was indeed part of LUBAC at the endogenous level. For this, we purified the endogenous LUBAC complex from human embryonic kidney 293ET (HEK293ET) cell lysates by gel filtration (Figure 7A). As reported previously (Kirisako et al., 2006), HOIP and HOIL-1 formed an approximately 600 kDa complex, and SHARPIN eluted quantitatively in this size range. The LUBAC complex is of similar size to recombinant p97 hexamers or to cellular p97 complexes. Bacterially purified OTULIN is monomeric and elutes according to its mass at ∼40 kDa. To our surprise, the majority of endogenous OTULIN in HEK293ET cells (>95%) eluted in a size range of ∼100–150 kDa, and only a small fraction seemed to coelute with the endogenous LUBAC complex (Figure 7A). Similar data were obtained in U2OS and RPE1 cells (Figure S8). This was in contrast to our findings that the HOIP PUB-OTULIN interaction was stable on gel filtration (Figure 1D). Although there were many potential reasons for why the interaction was unstable in cells, one intriguing possibility was that binding of OTULIN to HOIP was dynamically regulated. Indeed, OTULIN is phosphorylated in cells, and the prime site for phosphorylation is the PIM residue Tyr56 (http://phosphosite.org/proteinAction.do?id=2470471; Figure 7B). A Tyr56-phosphorylated PIM peptide was unable to bind HOIP, which is consistent with our structural data (Figure 7C). Importantly, the distribution of OTULIN changed significantly when HEK293ET lysates were prepared in the absence of phosphatase inhibitors. Although OTULIN eluted in a single peak when phosphatases are inhibited (Figure 7A), phosphatase activity resulted in two peaks at 600 and 40 kDa. This suggested that OTULIN is indeed phosphorylated in HEK293ET cell lysates and that dephosphorylation leads to quantitative association with LUBAC. OTULIN may be more abundant than LUBAC and HOIP, given that a significant fraction of dephosphorylated OTULIN is not bound to HOIP and elutes as a monomer. Altogether, this suggests that the abundance of OTULIN on LUBAC is regulated by phosphorylation of the OTULIN PIM.

## Discussion

Here, we reveal the molecular basis for the interaction of Met1-processing machineries, namely between the chain assembling LUBAC complex and the Met1-specific DUB, OTULIN. This is yet another example of interaction of a DUB with an E3 ligase in analogy to well-established complexes such as MDM2-USP7 (Li et al., 2004) or BRAP-USP15 (Hayes et al., 2012). What is unique about this complex is that all components are exquisitely specific for Met1-linked polyUb. The entire machinery appears to have coevolved to regulate this particular Ub chain type, and it is tempting to speculate that other chain types are regulated in a similar manner. We recently showed that OTU domain DUBs are highly linkage specific and include members with defined preference for rare atypical linkages (Mevissen et al., 2013). It will be interesting to see whether these DUBs associate with E3 ligases to form chain-type-specific processing complexes.

Our work assigns a function to the previously unstudied PUB domain of HOIP, which mediates the interaction with a short, conserved PIM in the OTULIN N terminus. PUB domains are found in only a handful of proteins that bind p97, including PNGase and UBXD1 (Allen et al., 2006; Suzuki et al., 2001). UBXD1 also contains a UBX domain and binds p97 via two interfaces (Kern et al., 2009). Interestingly, despite structural similarity and HOIP's ability to bind p97 peptides with similar affinity to other PUB domains, PNGase or UBXD1 cannot bind OTULIN. Moreover, PUB domains were not known to bind to internal sequences, and we show that a two-residue hydrophobic motif and a kink in the PIM peptide is necessary for interacting with PUB domains. This realization may lead to the identification of PIMs in other proteins and binding partners for PUB domain proteins, including HOIP. Although the shortness of the motif poses significant challenges to identifying PIMs by bioinformatic means, recent methods to predict that similarly short LC3-interacting motifs may be applicable (Kraft et al., 2012).

Despite its importance, the composition of the LUBAC complex is currently unclear. HOIP (120 kDa) and HOIL-1 (58 kDa) form a ∼600 kDa complex when purified from eukaryotic cells (Kirisako et al., 2006). Subsequently, SHARPIN (40 kDa) was shown to be an additional LUBAC component (Gerlach et al., 2011; Ikeda et al., 2011; Tokunaga et al., 2011) and was subsequently shown to dimerize (Stieglitz et al., 2012a). Here, we reveal that also SHARPIN participates in a 600 kDa LUBAC complex. Although all three proteins readily coimmunoprecipitate, suggesting a trimeric complex (see Emmerich et al., 2013), the gel filtration analysis does not exclude the presence of HOIP/HOIL-1 or HOIP/SHARPIN subcomplexes. Neither SHARPIN nor HOIL-1 can bind the PUB domain of HOIP (they contain one Tyr each and have no PIM), which would be free to interact with OTULIN or p97. We show that HOIP greatly favors OTULIN, and that p97 concentration must be rather high in order to compete with OTULIN if bound. However, given that p97 is a hexamer and HOIP is oligomeric, an interaction of complexes would most likely have improved binding properties.

Our study provides evidence that OTULIN regulates LUBAC-assembled Met1-polyUb through direct interaction with the HOIP PUB domain and that this might regulate LUBAC’s signaling capacity. Moreover, we show that endogenous OTULIN can be part of the endogenous LUBAC complex; however, this is prevented by the phosphorylation of the OTULIN PIM Tyr residue. The involved protein kinase(s) and phosphatase(s) and the dynamics of this phosphorylation event need additional investigation. The regulation of PUB-PIM interactions by phosphorylation was previously shown also for p97, in which PIM phosphorylation blocks PNGase interaction and affects endoplasmic-reticulum-associated protein degradation (Li et al., 2008). To fully understand the physiological consequences of the OTULIN-HOIP interaction, genetic models such as knockin animals or cell lines are required, and the dynamics of OTULIN phosphorylation need to be understood. Nonetheless, our characterization of OTULIN as a direct binding partner for LUBAC, and the realization that this interaction is regulated by phosphorylation, improves our understanding of the important Met1-polyUb-regulating machinery in cells and provides an elegant model as to how individual Ub chain types may be regulated by specific DUB-E3 pairs.

## Experimental Procedures

Additional details on all methods can be found in the Supplemental Experimental Procedures.

### Protein Expression and Purification

Proteins were expressed from pOPINB vectors in Rosetta2 (DE3) pLacI cells. For NMR studies, cells were grown in 2M9 medium supplemented with ^15^N NH_4_Cl and/or ^13^C glucose. Proteins were purified by immobilized metal-affinity, anion-exchange, and size-exclusion chromatography.

### Crystal Structure Analysis

Crystallization conditions were screened by the vapor diffusion method. Apo HOIP was determined by molecular replacement with SGC coordinates (PDB ID 4JUY) as a search model. The HOIP-OTULIN PIM structure was determined by molecular replacement with the apo HOIP structure.

### NMR Spectroscopy

Standard triple-resonance experiments (HNCA, HN(CO)CA, HNCACB, CBCA(CO)NH, and HBHA(CO)NH) were acquired for the assignment of HOIP resonances. Constant time ^13^C and ^13^C-HSQC were acquired for the methyl and aromatic regions. In addition, (HB)CB(CGCD)HD and (HB)CB(CGCDCE)HE experiments coupled the Cβ of tyrosine resonances to the Hδ and Hε positions of the tyrosine ring, respectively.

### Fluorescence Polarization Binding Assays

Serially diluted PUB domains and HOIP variants were mixed with an equal volume of 100 nM FITC-Ahx-labeled peptides of OTULIN and p97. Fluorescence polarization was recorded on a PheraStar plate reader (BMG LABTECH) and fitted to a one-site binding model with GraphPad Prism 5.

### Immunoprecipitation of HOIP-V5 and HA-OTULIN

Transfected HEK293T or U2OS and NOD2 cells were lysed in the presence of protease and phosphatase inhibitors. Clarified lysates were incubated overnight with anti-V5 and anti-HA-agarose resin.

### Luciferase Reporter Assays

Cells were cotransfected with the NFκB luciferase reporter construct pBIIXluc and the thymidine kinase-renilla luciferase construct in addition to other vectors used in the study. After 24 hr, cells were lysed in passive lysis buffer (Promega), and luciferase activity was recorded. Protein expression levels were determined by western blotting of cell lysates.

## Figures and Tables

**Table 1:** Data Collection Statistics

	HOIP 1–184	HOIP 5–180 + OTULIN 49–67
**Data Collection**		

Beamline	Diamond I04	Diamond I02
Space group	C *2*	*P 6_1_*
*a*, *b*, *c* (Å)	155.20, 99.57, 173.66	64.05, 64.05, 172.02
α, β, γ (°)	90.00, 99.88, 90.00	90.00, 90.00, 120.00
Wavelength	0.9794	0.9795
Resolution (Å)	65.75-3.00 (3.09-3.00)	55.47-2.00 (2.05-2.00)
*R*_merge_	12.6 (45.1)	12.9 (60.4)
*I* / σ*I*	6.9 (2.3)	5.9 (2.0)
Completeness (%)	99.6 (99.5)	99.9 (99.8)
Redundancy	2.8 (2.8)	4.9 (5.2)

**Refinement**		

Resolution (Å)	62.61-3.00	55.47-2.00
Number of reflections	52,158	26,676
*R*_work_ / *R*_free_	21.8 (25.8)	20.2 (23.5)
Number of atoms		
Protein	18,228	3,262
Ligand/ion	150	3
Water	16	281

**B factors**		

Wilson *B*	36.0	22.4
Protein	20.2	29.8
Ligand/ion	43.2	36.8
Water	15.2	32.8

**rmsd**		

Bond lengths (Å)	0.003	0.002
Bond angles (°)	0.740	0.613
Ramachandran statistics (favored/allowed/outliers)	97.65/2.26/0.09	98.38/1.62 /0.0

Numbers in brackets are for the highest-resolution shell.

## Accession Numbers

Coordinates and structure factors have been deposited to the PDB under accession numbers 4OYJ (HOIP-PUB domain) and 4OYK (HOIP-PUB in complex with OTULIN PIM).

## Supplemental Information

Document S1. Supplemental Experimental Procedures and Figures S1–S8

Document S2. Article plus Supplemental Information
