# Supplementary material for: Molecular Basis and Regulation of OTULIN-LUBAC Interaction
Source: Mol Cell. 2014 May 8;54(3):335–48. doi: 10.1016/j.molcel.2014.03.018 (PMC4017264; doi:10.1016/j.molcel.2014.03.018)
Supplement: Document S1. Supplemental Experimental Procedures and Figures S1–S8 [file mmc1.pdf]

Molecular Cell, Volume *53*

Supplemental Information

Molecular Basis and Regulation of OTULIN-LUBAC Interaction

Paul R. Elliott, Sofie V. Nielsen, Paola Marco-Casanova, Berthe Katrine Fiil, Kirstin Keusekotten, Niels Mailand, Stefan M.V. Freund, Mads Gyrd-Hansen, and David Komander

Supplementary Figure 1

A

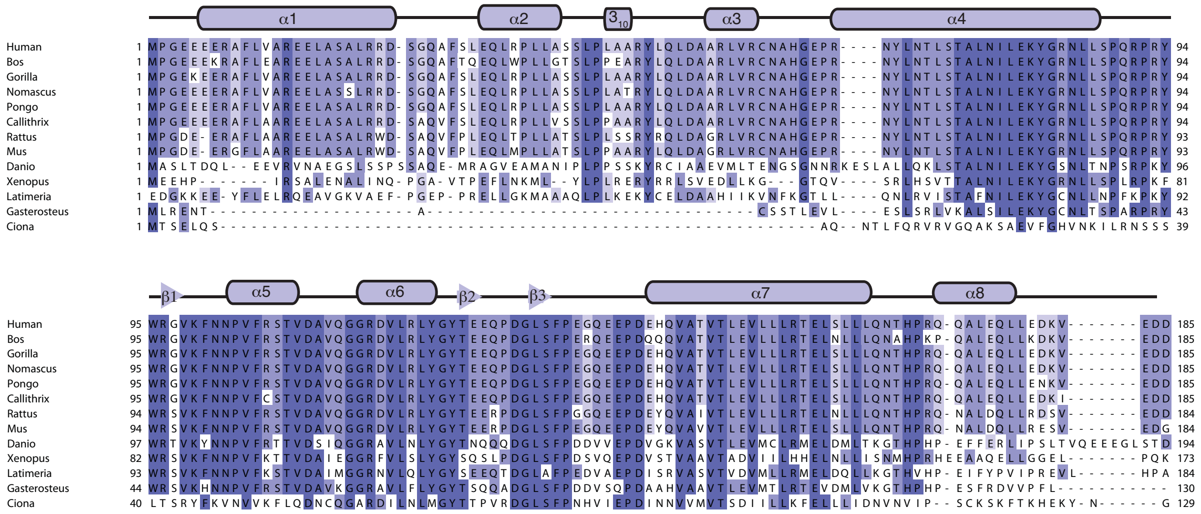

B

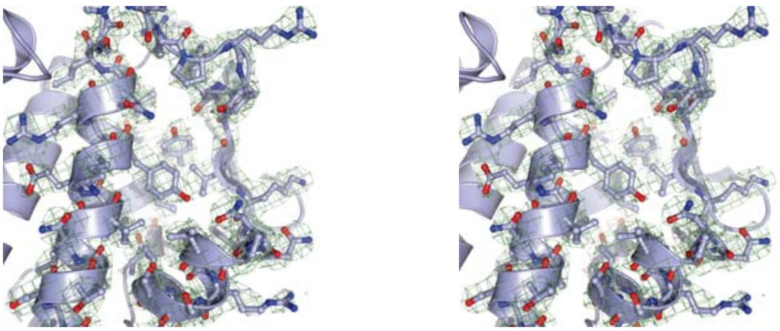

C

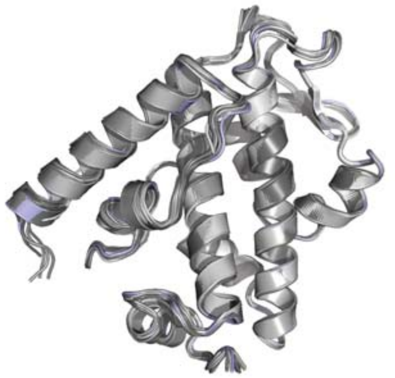

D

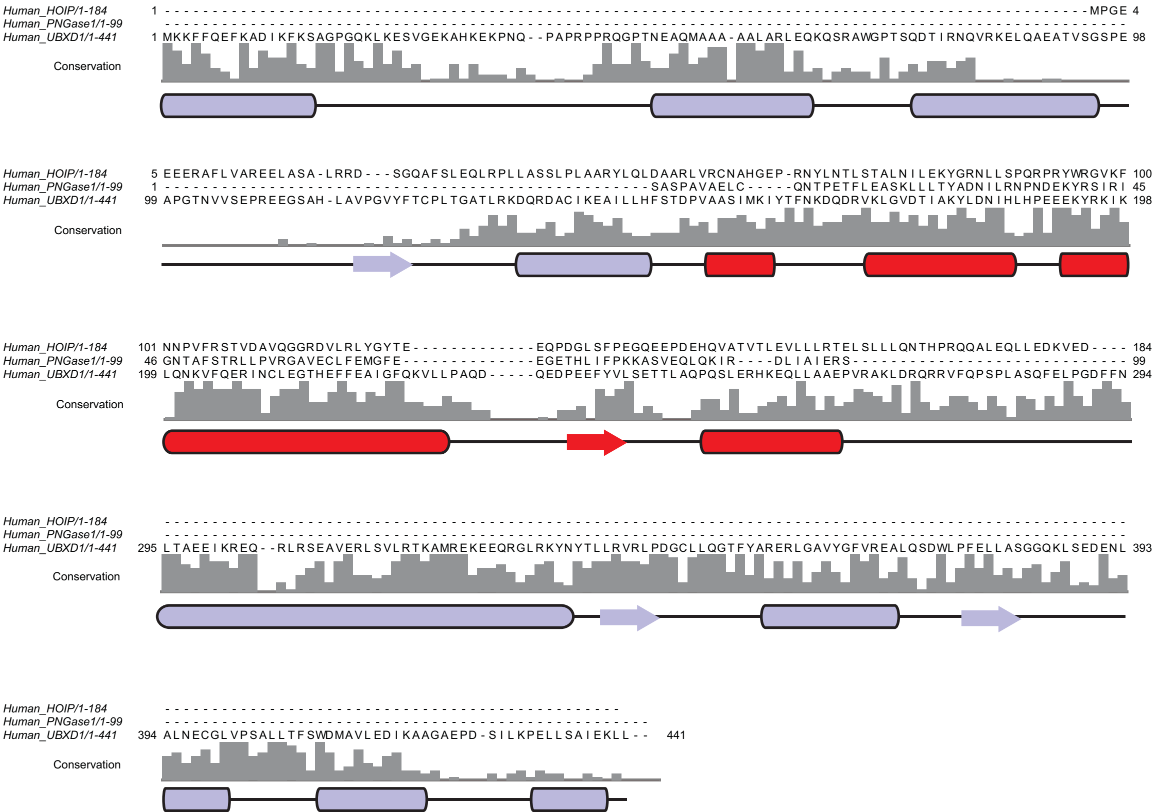

Supplementary Figure 1 (linked to Figure 1)

A) Multiple sequence alignment of HOIP PUB domain orthologs, extracted from the Ensemble Genome Browser. Secondary structure annotations corresponding to the HOIP PUB domain structure are shown above the sequence alignment. B) Cross-eyed stereo pair of apo HOIP showing residues around the PIM pocket. Residues are shown in ball and stick representation enclosed in a weighted 2|Fo|-|Fc| map contoured at 1 σ. C) Superimposition of the 13 HOIP PUB domain molecules observed within the asymmetric unit of the apo HOIP PUB domain structure. All chains superimpose with low RMSDs (0.9-1.2 Å). D) UBXD1 PUB domain does not contain an N-terminal extension as found in HOIP PUB domain. Sequence alignment of the HOIP and PNGase PUB domains onto UBXD1 full-length sequence. Sequence conservation of UBXD1 orthologs is shown as bar graphs. Predicted secondary structure elements for human UBXD1 are shown. The region corresponding to the annotated PUB domain is colored in red. In UBXD1 there is an area of low conservation N-terminal to the annotated PUB domain, suggesting lack of any conserved N-terminal domain, in contrast to the HOIP PUB domain.

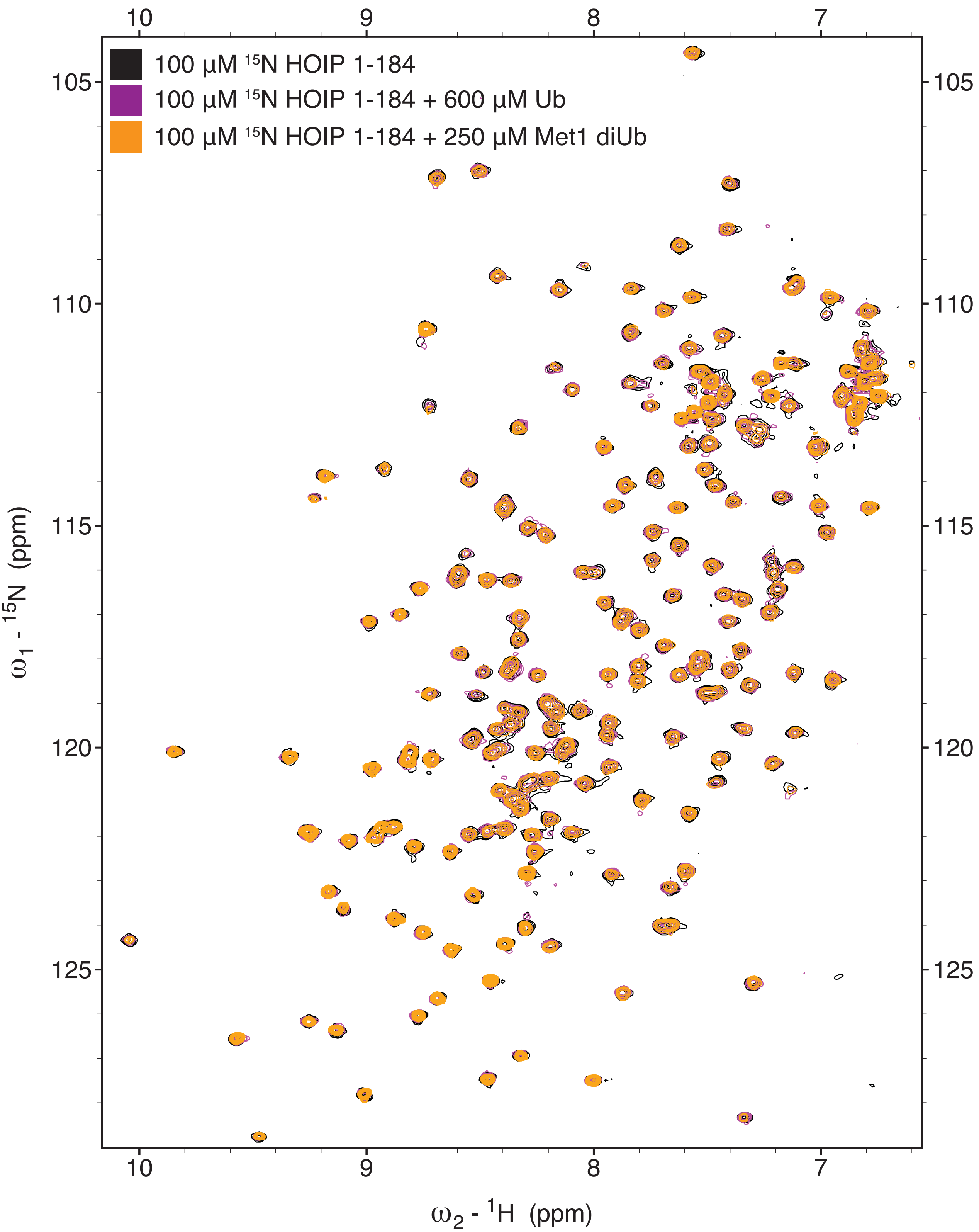
100 µM 15N HOIP 1-184
100 µM 15N HOIP 1-184 + 600 µM Ub
100 µM 15N HOIP 1-184 + 250 µM Met1 diUb
10
9
8
7
105
110
115
120
125
ω1 - 15N (ppm)
ω2 - 1H (ppm)

Supplementary Figure 2 (linked to Figure 2A, B)

Heteronuclear Single Quantum Correlation (HSQC) spectra of ^{15}N-labeled HOIP (aa 1-184) (black) with six molar excess of ubiquitin (magenta) or two and a half molar excess of Met1-diUb (orange) are shown. No chemical shift perturbations were observed upon addition of Ub or Met1-diUb, suggesting that the HOIP PUB domain does not contain a Ub interaction surface, in contrast to PNGase (Kamiya et al., 2012).

Supplementary Figure 3

A

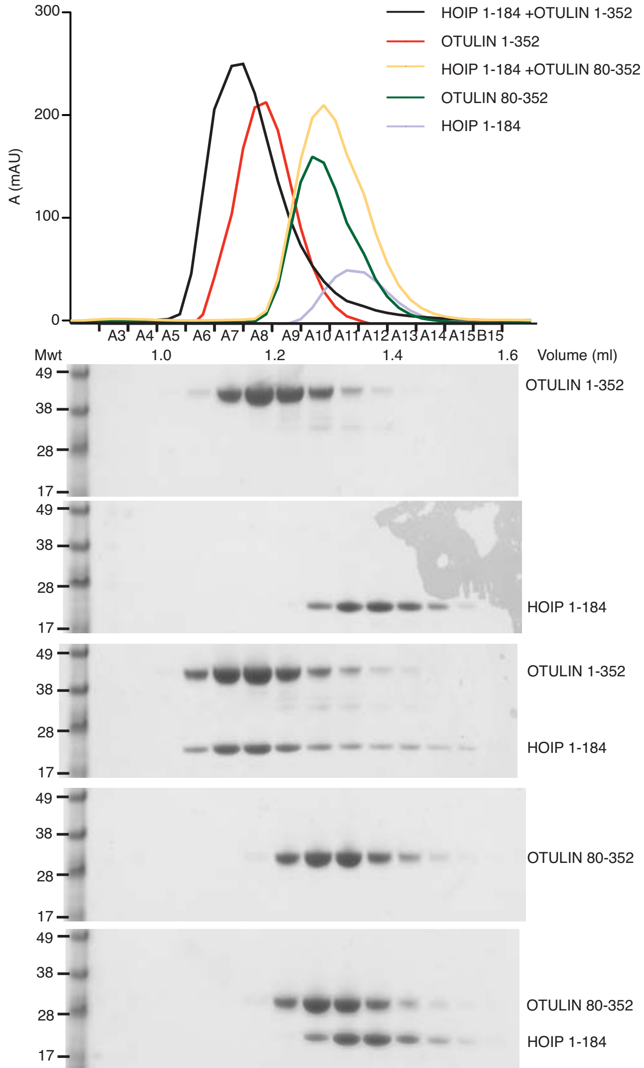

B

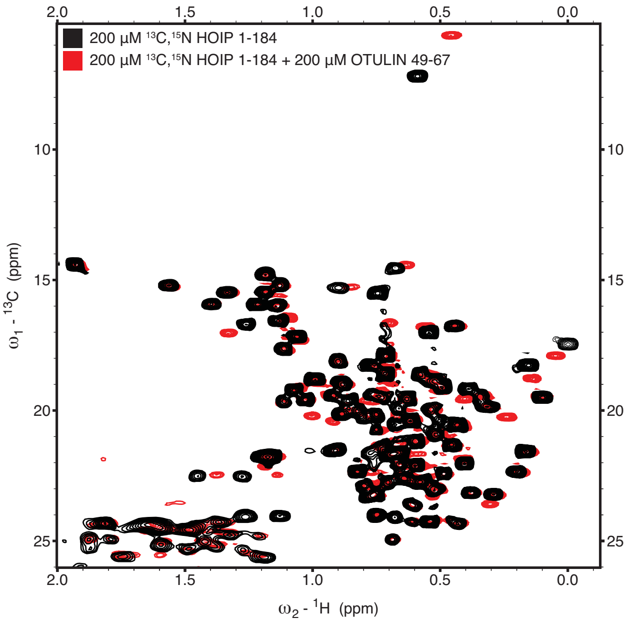

Supplementary Figure 3 (linked to Figure 2C-G)

A) Analytical size exclusion chromatography profile of the HOIP 1-184 (blue), full-length OTULIN 1-352 (red), 1.2:1 complex of HOIP 1-184 and OTULIN 1-352 (black) (as shown in **Figure 1E**). OTULIN catalytic domain 80-352 (green) and OTULIN 80-352 mixed with HOIP 1-184 in a 1:1 molar ratio (yellow). Coomassie-stained SDS-PAGE gels are shown below for the protein-containing fractions. B) Aliphatic-region of ^{13}C-HSQC spectra for HOIP 1-184 (black) and HOIP-1-184 in a 1:1 complex with OTULIN PIM 49-67 (red). In contrast to the ^{15}N-BEST TROSY experiments, only a small subset of chemical shift perturbations are observed upon OTULIN PIM binding. This is in agreement with a small interacting region on the HOIP PUB domain, consistent with the HOIP-OTULIN PIM crystal structure.

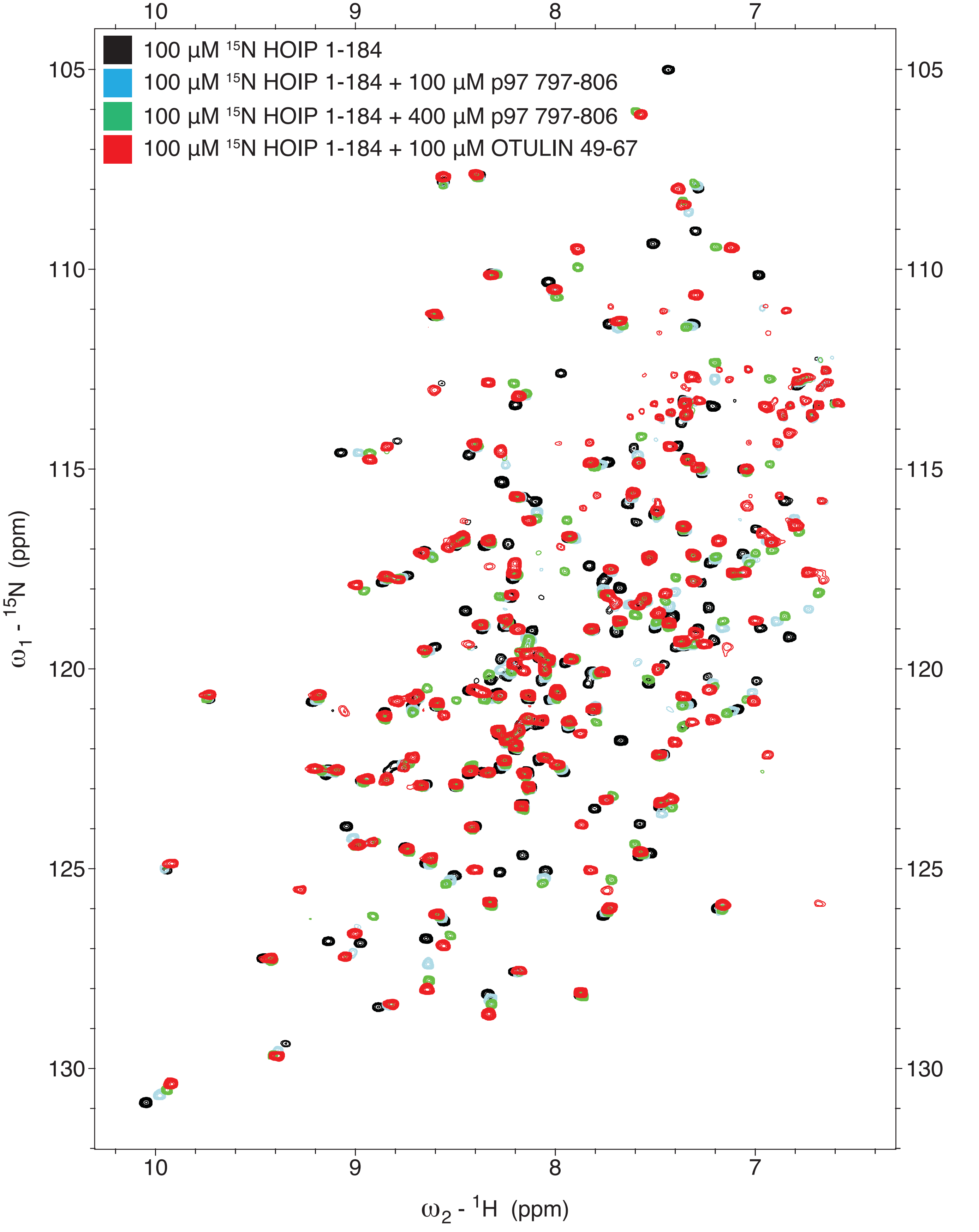
100 µM ¹⁵N HOIP 1-184
100 µM ¹⁵N HOIP 1-184 + 100 µM p97 797-806
100 µM ¹⁵N HOIP 1-184 + 400 µM p97 797-806
100 µM ¹⁵N HOIP 1-184 + 100 µM OTULIN 49-67
ω1 - 15N (ppm)
ω2 - 1H (ppm)
10
9
8
7
105
110
115
120
125
130

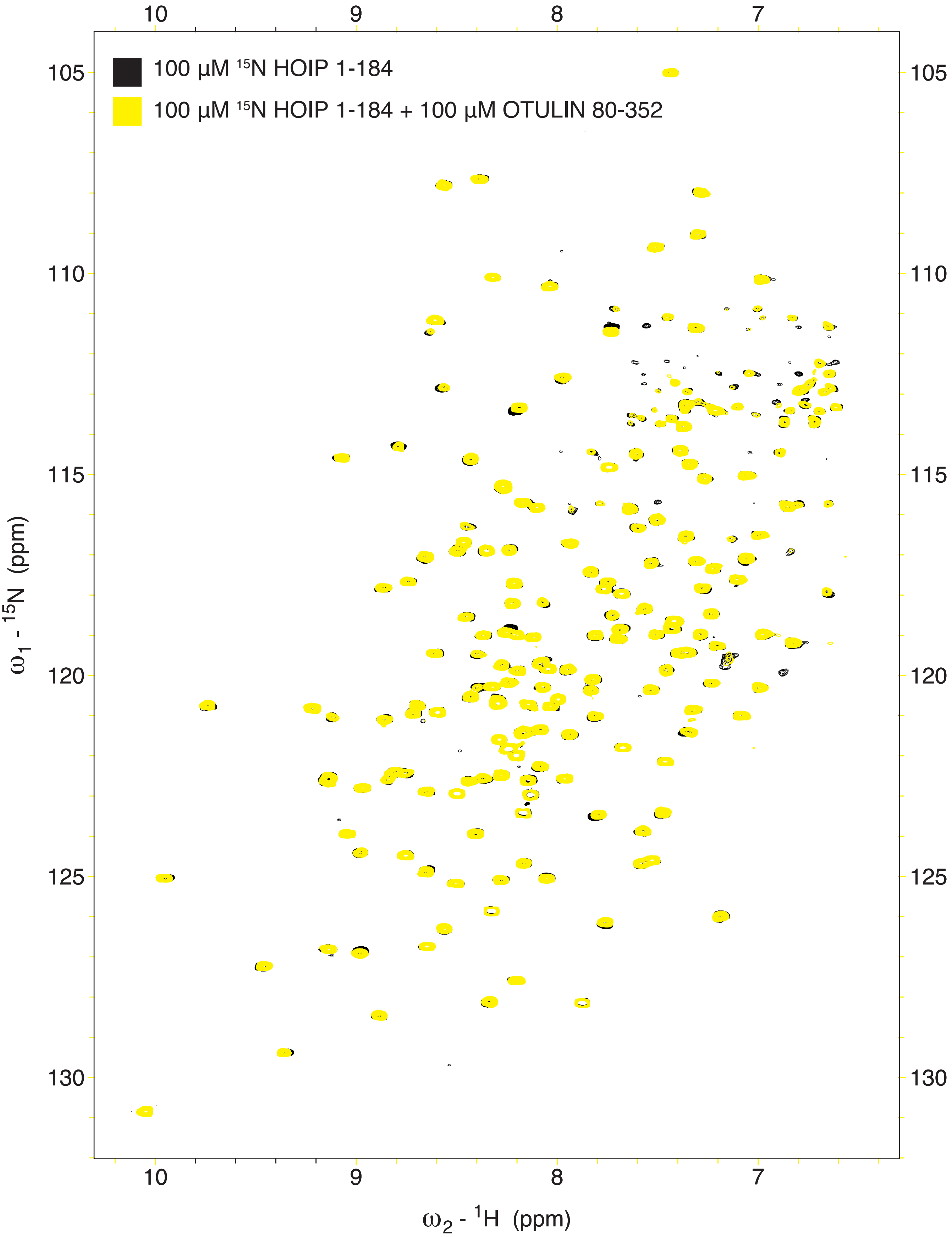
100 μM 15N HOIP 1-184
100 μM 15N HOIP 1-184 + 100 μM OTULIN 80-352
10
9
8
7
105
110
115
120
125
130
ω1 - 15N (ppm)
ω2 - 1H (ppm)

Supplementary Figure 4C

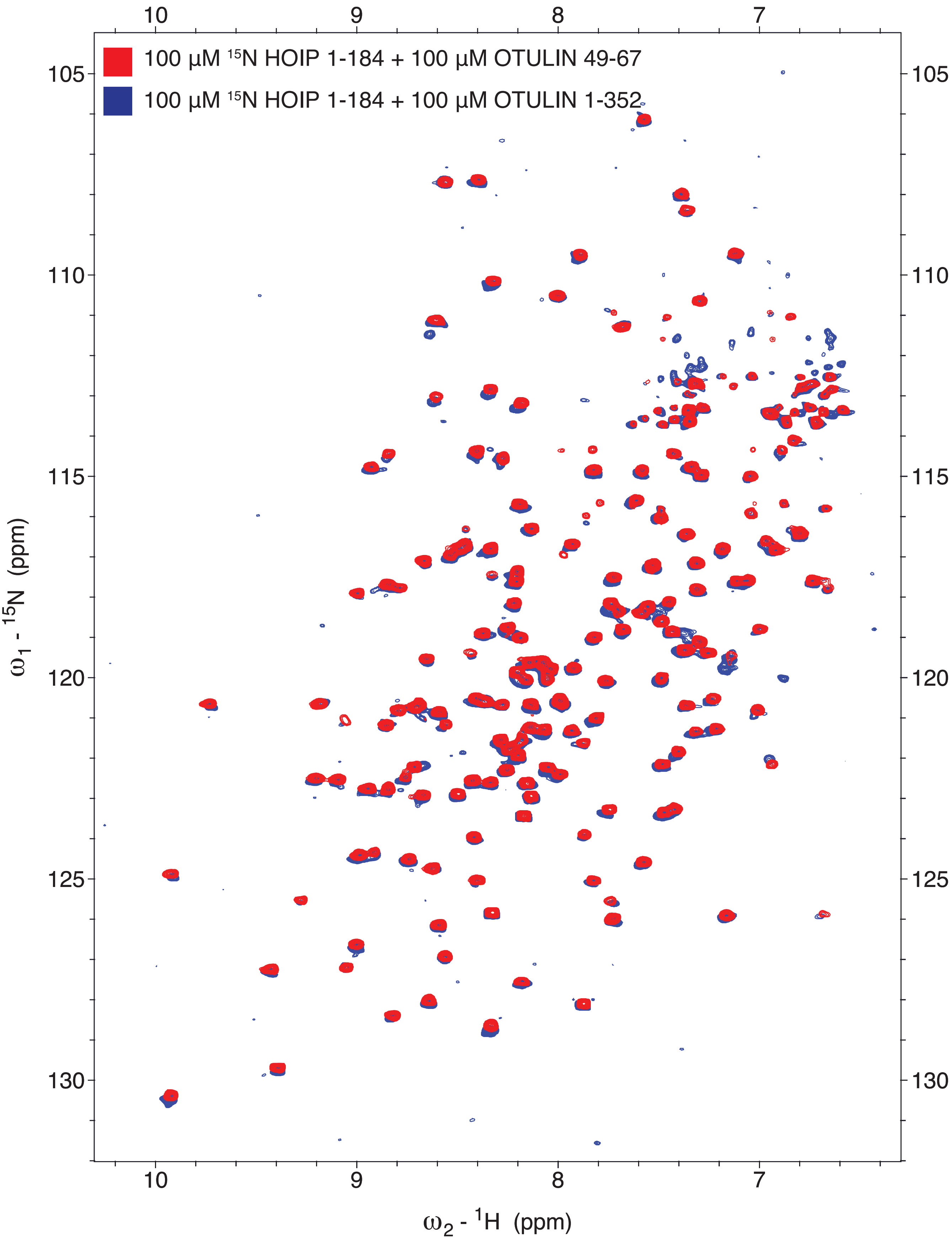

Supplementary Figure 4 (linked to Figure 2G-J)

^{15}N-BEST TROSY spectra for A) ^{15}N-labeled HOIP (black) mixed with an equimolar amount (blue) or four fold excess (green) of unlabeled p97 peptide, or mixed with an equimolar amount of OTULIN peptide (red). B) ^{15}N-labeled HOIP (black) mixed with an equimolar amount of unlabeled OTULIN OTU domain (yellow). C) ^{15}N-labeled HOIP mixed with an equimolar amount of unlabeled OTULIN peptide (red) or full-length OTULIN (blue).

Supplementary Figure 5

A

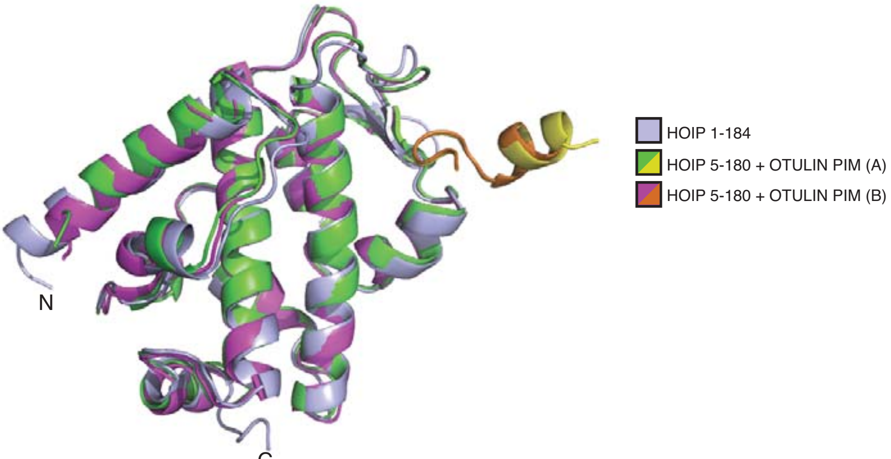

B

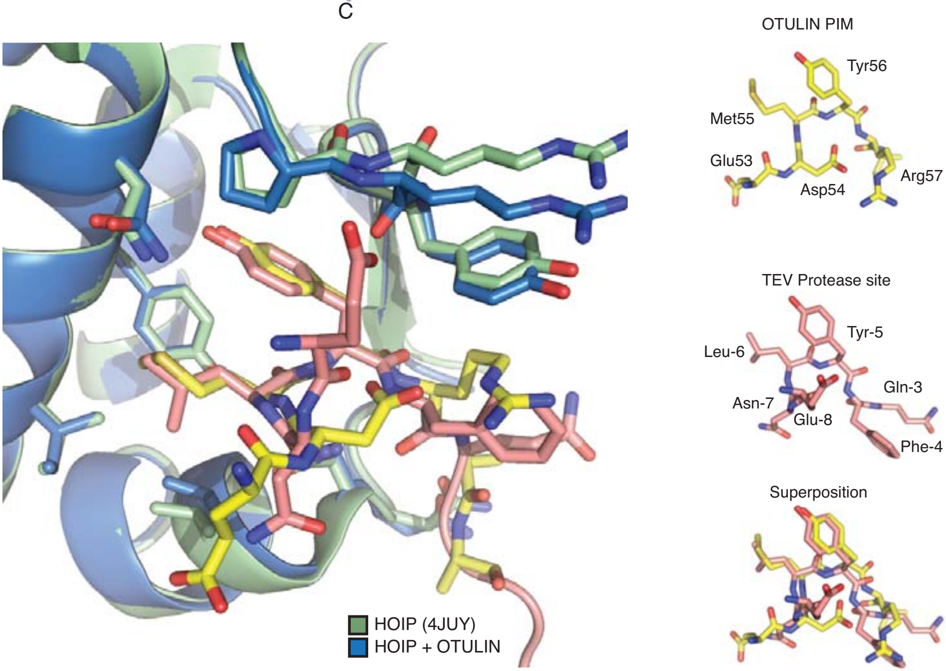

Supplementary Figure 5 (linked to Figure 3)

A) Superimposition of the HOIP-OTULIN PIM complex from the asymmetric unit (green PUB domain with yellow OTULIN PIM, and magenta PUB with orange OTULIN PIM) onto apo HOIP (light blue). Two molecules of the HOIP-OTULIN PIM complex are observed in the asymmetric unit and shown superimposed onto each other with low RMSD (1.01 Å), both superimpose well onto the apo HOIP with an RMSD of 1.51 Å. B) Superimposition of the HOIP PUB domain (blue) with the HOIP PUB domain determined by the SGC (pdb-id 4juy, green). In both structures Tyr94 is flipped out, compared to apo HOIP structure where it partially occludes the PIM binding site. The SGC structure was crystallized with the His6 tag present. The TEV site forms a putative PIM (EDLYQ) (red) compared to the OTULIN PIM (DEMYR) (yellow) and superimpose well (right). In the SGC HOIP structure, the putative PIM from the TEV site in the symmetry-related molecule packs against the OTULIN PIM binding site on HOIP, resulting in Tyr94 been stabilized as in the holo state.

A

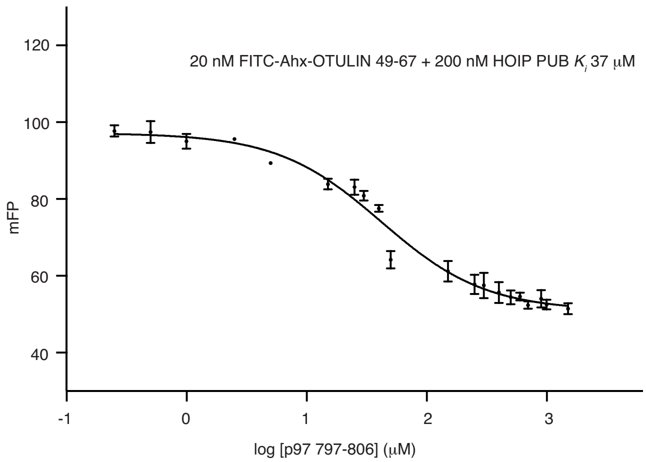
20 nM FITC-Ahx-OTULIN 49-67 + 200 nM HOIP PUB K_i 37 μM
120
100
80
60
40
mFP
-1
0
1
2
3
log [p97 797-806] (μM)

B

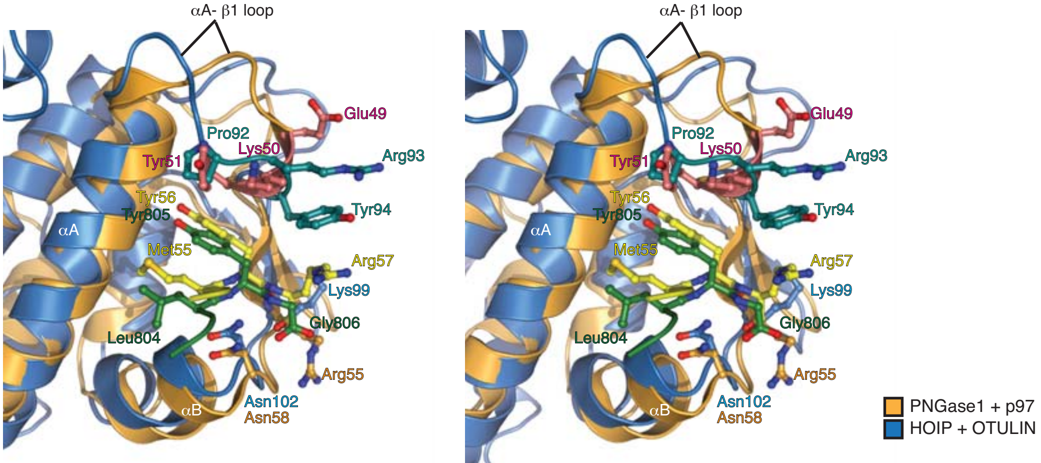
αA- β1 loop
αA- β1 loop
Glu49
Pro92
Lys50
Arg93
Tyr51
Tyr56
Tyr805
Tyr94
αA
Met55
Arg57
Lys99
Gly806
Leu804
Arg55
Asn102
Asn58
αB
PNGase1 + p97
HOIP + OTULIN

Supplementary Figure 6 (linked to Figure 4)

A) Competition assay using fluorescent OTULIN PIM (49-67) and unlabeled p97 PIM (797-806). Wells containing fixed concentration of HOIP (200 μM) bound to 20 nM FITC-Ahx OTULIN PIM are mixed with varying concentrations of unlabeled p97 PIM and the decrease in fluorescent polarization is monitored. Experiments were performed in triplicate and errors represent standard deviation from the mean. The data is fitted against a one-site model for determining K_i in GraphPad Prism. B) Cross-eyed stereo pair of the superimposed HOIP and PNGase PUB domains. The OTULIN PIM peptide sits deeper into the HOIP PIM pocket, relative to the p97 PIM peptide bound to PNGase.

Supplementary Figure 7

A

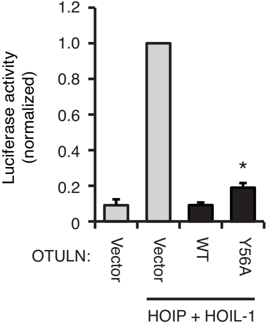

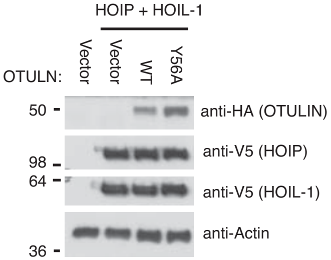

Supplementary Figure 7 (linked to Figure 6)
A) NFκB reporter assay performed as in **Figure 6D** for OTULIN Y56A PIM mutant co-transfected in HEK293T cells with epitope-tagged HOIP and HOIL1. Luciferase reporter activity for the OTULIN Y56A PIM was marginally greater than that of transfected wt OTULIN. *Right*, corresponding Western-Blot analysis of the assay.

Supplementary Figure 8

A

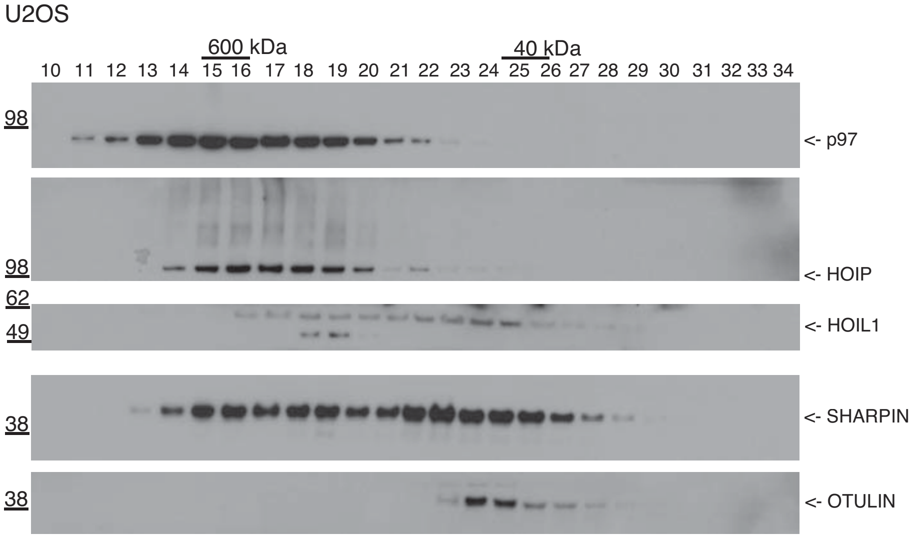

B

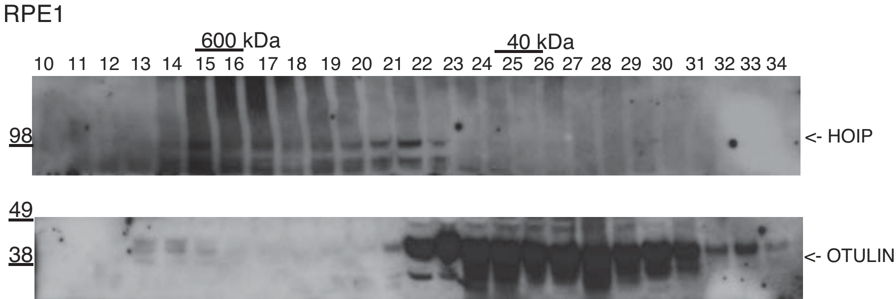

Supplementary Figure 8 (linked to Figure 7)

A) Gel filtration analysis of unstimulated U2OS cells, performed as in **Figure 7A**. B) Gel filtration analysis of human primary retinal pigment epithelial cells (RPE1), blotted for HOIP and OTULIN.

Supplementary Experimental Procedures

Sequence analysis

Multiple alignment analysis was performed with ClustalX. For sequences of the OTULIN N-terminal region used for analysis, see Supporting Information. Species abbreviations are as follows: Hs (*Homo sapiens*), Mam (*Macaca mulatta*), Mm (*Mus musculus*), Clf (*Canis lupus familiaris*), Fc (*Felis catus*), Bt (*Bos taurus*), Eq (*Equus caballus*), Ss (*Sus scrofa*), Tg (*Taeniopygia guttata*), Sas (*Salmo salar*), Dr (*Danio rerio*), Xt (*Xenopus tropicalis*), Dm (*Drosophila melanogaster*) Ce (*Caenorhabditis elegans*), Sc (*Saccharomyces cerevisiae*).

Molecular biology

pcDNA3-HOIP-V5/His, pcDNA3-HOIPΔPUB+ZnF-V5/His and pcDNA3-HOIL-1-V5/His were generously supplied by Prof. Henning Walczak (University College London, London, UK). The NFκB luciferase reporter plasmids, pBIIX-Luc and TK-renilla-Luc have been described previously (Gyrd-Hansen et al., 2008). HOIP-PUB (amino acid residues 1-185) and HOIP-PUB+ZnF (amino acid residues 1-436) fragments were amplified by PCR using the following primers *AAAGGTACC*ATGCCGGGGGAGGAAGAGG / *AACTCGAG*ATCATCTTCAACCTTGTCTTCC and *AAAAAGCTTG*ATGCCGGGGGAGGAAGAGG / *AATCTAGA*ACTAGTCCGGTTGCACATAAC, and were inserted into pcDNA3-V5/His-A. The Glutathione-S-transferase (GST) M1-SUB construct has been described previously (Fiil et al., 2013; Keusekotten et al., 2013). All constructs have been verified by DNA sequencing.

For biochemical and structural studies, the coding sequence for the HOIP PUB domain was amplified using KOD HotStart DNA polymerase using the following primers: HOIP-1-Fwd AAGTTCTGTTTCAGGGCCCGATGCCGGGGGAGGAAGAG and HOIP-184 Rev ATGGTCTAGAAAGCTTTAATCTTCAACCTTGTCTTCCAACAG. The PCR product was cloned into pOPINB, which encodes a 3C cleavable N-terminal His6-tag (Berrow et al., 2007) using Infusion HD cloning (Clontech).

All mutations were generated by site directed mutagenesis using the QuikChange method with KOD HotStart DNA polymerase.

Protein expression and purification

HOIP PUB domain constructs were expressed in Rosetta2 (DE3) pLacI cells. Cells were grown at 30 °C in 2xTY medium supplemented with 30 µg/ml kanamycin and 34 µg/ml chloramphenicol to an OD600 of 0.8. The culture was cooled to 18 °C prior to overnight induction with 400 µM IPTG. Cells were resuspended and lysed by sonication in lysis buffer (20 mM Tris pH 7.4, 300 mM NaCl, 50 mM imidazole, 2 mM β-mercaptoethanol, lysozyme, DNaseI (Sigma) and protease inhibitor cocktail (Roche)). HOIP was purified by immobilized metal affinity chromatography using a HisTrap column (GE Life Sciences). The His6-tag was cleaved by overnight incubation with 3C protease. The protein was buffer exchanged into 20 mM Tris pH 8.5, 2 mM DTT and purified further by anion exchange chromatography (ResourceQ, GE Life Sciences). Eluted HOIP was then subjected to size exclusion chromatography (HiLoad 16/60 Superdex 75, GE Life Sciences) in buffer containing 20 mM Tris pH 8.5, 150 mM NaCl, 2 mM DTT. The resultant fractions were judged to be 99% pure following SDS-PAGE analysis and flash frozen. OTULIN was expressed and purified according to (Keusekotten et al., 2013). Human PNGase (aa 11-109) and UBXD1 (aa 150-264) proteins were a kind gift from Mark Allen (MRC LMB).

Analytical size exclusion chromatography binding studies

Binding studies using HOIP PUB domain and variants of OTULIN were performed on an AKTA Micro system (GE Life Sciences) using a Superdex 75 PC 3.2/30 column equilibrated SEC buffer (20 mM Tris pH 7.4, 150 mM NaCl, 2 mM DTT). HOIP PUB was mixed with OTULIN in a 1.2 : 1 molar ratio (30 µM HOIP and 25 µM OTULIN) and incubated at room temperature for 15 min. Fractions containing protein were mixed with SDS loading buffer prior to SDS-PAGE analysis.

Crystallization

Crystals of the HOIP PUB domain alone were grown by hanging-drop vapor diffusion. HOIP PUB was mixed with an equal volume of reservoir (1.3 M ammonium sulfate, 100 mM Tris (pH 8.5), 200 mM KI). Two crystal forms appeared over the course of two weeks; rod-shaped crystals that diffracted poorly and small rounded crystals that were used for diffraction experiments. Crystals were transferred into 3.5 M ammonium sulfate and vitrified prior to data collection. For crystallization of the HOIP-OTULIN peptide complex, a shorter construct of HOIP was used (5-180) that omitted terminal residues not observed in the electron density of the apo HOIP structure. HOIP 5-180 was mixed with a 1.5 molar excess of OTULIN peptide (49-67). Crystals were grown by sitting-drop vapor diffusion. HOIP OTULIN peptide complex were mixed with reservoir containing 32% PEG 6000, 1 M LiCl, 100 mM Tris (pH 8.4) in a 1:2 ratio. Crystals were transferred to a solution containing 34% PEG 6000, 1 M lithium chloride, 100 mM Tris (pH 8.4) prior to cryo-cooling.

Data collection, structure determination and refinement

Diffraction data were collected at Diamond Light Source beam lines I02 and I04. Diffraction images were processed using MOSFLM (Battye et al., 2011) and scaled using AIMLESS (Winn et al., 2011). The structure of apo HOIP was determined by molecular replacement using PHASER (McCoy et al., 2007) using the HOIP PUB domain structure deposited by the SGC (pdb-id 4juy) as a search model. 13 molecules of HOIP PUB domain were found within the asymmetric unit. Iterative rounds of model building and refinement were performed with COOT (Emsley et al., 2010) and PHENIX (Adams et al., 2011), respectively. The HOIP-OTULIN peptide structure was also determined by molecular replacement using a single HOIP PUB domain as a search model. Following model building and refinement the OTULIN peptide could be built unambiguously into the electron density. Data collection and refinement statistics can be found in **Table 1**. All structural figures were generated with Pymol (www.pymol.org).

Fluorescence polarization binding assays

10 µl of 100 nM FITC-Ahx peptides of either OTULIN (49-67) or p97 (797-806) were aliquoted into a 384-well low volume plate (Corning). Serial dilutions of HOIP PUB into FP assay buffer (20 mM Tris pH 7.4, 100 mM NaCl, 1 mM DTT) were prepared and 10 µl of this was aliquoted to FITC-Ahx peptide-containing wells. Fluorescence polarization was recorded on a PheraStar plate reader (BMG Labtech) using an optics module with λ_{ex} = 485 nm and λ_{em} = 520 nm. To derive binding constants (K_D), polarization values were fitted to a one-site binding model using Graphpad Prism 5.

Nuclear Magnetic Resonance Spectroscopy

Uniformly labeled ^{15}N or ^{13}C, ^{15}N samples of HOIP were prepared following growth in 2M9 medium supplemented with either ^{15}N NH_4Cl or ^{13}C glucose. Proteins were purified as described above and all samples were exchanged into NMR sample buffer (20 mM Na_2HPO_4/NaH_2PO_4 pH 6.8, 50 mM NaCl, 2 mM DTT). Lyophilized OTULIN and p97 PIM peptides were reconstituted in NMR sample buffer and the pH adjusted to pH 6.8 prior to use. NMR data were acquired at 298K on Bruker Avance III 600 MHz and Avance2+ 700 MHz spectrometers, equipped with cryogenic triple resonance TCI probes. Standard triple resonance experiments (HNCA, HN(CO)CA, HNCACB, CBCA(CO)NH, HBHA(CO)NH) were acquired for the assignment of backbone HOIP resonances. In addition, constant-time (ct) ^{13}C and ^{13}C-HSQC were acquired for the methyl and aromatic regions. For assignment of the Hδ,Hε resonances of HOIP tyrosines, (HB)CB(CGCD)HD and (HB)CB(CGCDCE)HE spectra were acquired, which coupled the Cβ position of the tyrosine resonances to the Hδ/Hε position of the tyrosine ring respectively. Data processing and analysis were performed with Topspin3.0 (Bruker) and Sparky (http://www.cgl.ucsf.edu/home/sparky/).

RNA interference

Reverse transfection of HEK293T cells with siRNAs was performed using Lipofectamine RNAiMAX (Invitrogen) according to the manufacturer's

instructions. The following siRNA oligonucleotides (Sigma-Aldrich) were used for RNAi-mediated knockdown of OTULIN:
siOTULIN: GACUGAAAUUUGAUGGGAA, siMM
GGGAUACCUAGACGUUCUA.

Receptor stimulation

U2OS/NOD2 cells were treated with 200 ng/mL NOD2 ligand L18-MDP (InvivoGen) or 10 ng/mL TNFα (R&D systems) for the indicated times. Both were added directly to the culture medium.

Immunoprecipitation of HOIP-V5 and HA-OTULIN

HEK293T or U2OS/NOD2 cells were transfected as indicated. The cells were lysed in IP buffer buffer (25 mM HEPES pH 7.4, 150 mM KCl, 2 mM $MgCl_2$, 1 mM EGTA, 0.5% (v/v) Triton X-100) supplemented with 5 mM NEM (Sigma-Aldrich), cOmplete protease inhibitor cocktail (Roche) and PhosSTOP (Roche). After 30 minutes on ice, the lysates were cleared by centrifugation and incubated overnight at 4 ^{o}C with anti-V5-agarose resin or anti-HA-agarose resin. The beads were washed four times in ice-cold IP buffer and the precipitated material was eluted with 0.2 M glycine, pH 2.5.

Luciferase reporter assays

Cells were co-transfected with the NFκB luciferase reporter construct pBIIXluc and the thymidine kinase-renilla luciferase construct to enable normalization of transfection efficiency. Cells were co-transfected with additional plasmids as indicated. After 24 h cells were lysed in 75 µL passive lysis buffer (Promega) and luciferase activity was measured on a FLUOstar Omega Microplate Reader (BMG LABTECH GmbH, Offenburg, Germany) using the DualLuciferase ® Assay System (Promega) according to the manufacturer's instructions. Protein expression levels were determined by SDS-PAGE and Western blotting of cell lysates as indicated. The data shown represents mean ± SEM. A two-tailed Student´s t-test was applied to evaluate statistical significance.

Antibodies and affinity resin

The following antibodies and reagents were used according to the manufacturers' instructions: anti-HA-agarose resin (A2095), anti-V5-agarose resin (A7345) and rabbit polyclonal anti-HOIP/RNF31 (SAB2102031, Sigma-Aldrich), rat monoclonal anti-HA (#11867423991, Roche Diagnostics, Burgess Hill, UK), mouse monoclonal anti-ubiquitin (IMG-5021, Imgenex, San Diego, CA), rabbit polyclonal anti-SHARPIN (#14626-1-AP, ProteinTech, Chicago, IL), rabbit polyclonal anti-RBCK1/HOIL-1 (NBP1-88301, Novus Biologicals, Littleton, CO), mouse monoclonal anti-V5 (MCA1360, AbD Serotec, Kidlington, UK), mouse monoclonal anti-β-actin (MAB1501, Chemicon, Millipore, Billerica, MA), rabbit polyclonal anti-HOIL-1, rabbit polyclonal anti-HOIP/RNF31 (Sigma), mouse monoclonal anti-p97 (Thermo Scientific). The Fam105B/OTULIN antibody was previously described (Keusekotten et al., 2013). HRP-conjugated rabbit polyclonal anti-mouse IgG (P026002-2, Dako, Glostrup, Denmark), HRP-conjugated polyclonal goat anti-rabbit IgG (PI-1000, Vector Laboratories), HRP-conjugated goat polyclonal anti-rat IgG (#31470; Pierce, Thermo Scientific).

Cell lines

HEK293T and U2OS-Flp-In™ T-REx™ (U2OS/NOD2) were cultured as previously described (Damgaard et al., 2012; Fiil et al., 2013) and transfected using FuGeneHD and FuGene6 (Promega, Promega Corporation, Madison, WI, USA), respectively.

Purification of endogenous Met1-polyUb conjugates

Met1-polyUb conjugates were precipitated from U2OS/NOD2 cells using affinity reagents. For isolation of Met1-Ub chains, recombinant UBAN-GST fusion protein (M1-SUB) was used as described previously (Damgaard et al., 2013; Fiil et al., 2013). Briefly, the cells were lysed in buffer (20 mM Na_2HPO_4, 20 mM NaH_2PO_4, 1% NP-40, 2 mM EDTA) supplemented with 5 mM NEM (Sigma-Aldrich), cOmplete protease inhibitor cocktail (Roche), PhosSTOP (Roche) and 100 mg/mL M1-SUB. Lysates were cleared by centrifugation, mixed with Glutathione Sepharose 4B beads (GE Healthcare) and incubated

at 4 ºC for a minimum of 2 h with rotation. Beads were washed five times in 500 mL ice-cold PBS Tween-20 (0.1%). Precipitated material was eluted with 1 x LSB-buffer.

Size exclusion chromatography analysis of cell extracts

HEK293ET, RPE1 and U2OS cells were collected, washed with PBS and lysed on ice for 30 minutes in SEC cell extraction buffer (150 mM NaCl, 50 mM Tris-HCl pH 7.4, 1 mM $MgCl_2$, 1 mM DTT, 0.3% NP40, protease and (Roche)). Phosphatase inhibitors (Roche) were added unless otherwise stated. Lysates were centrifuged at 10,0000 x g for 30 minutes and subsequently filtered with a 2 µm-pore minispin column pack (Generon). In total 3.1 mg protein extract was analyzed on a Superdex 200 10/300 GL column (GE Life Sciences). 0.5 mL fractions were collected concentrated by TCA precipitation. One twelfth of each fraction was loaded and resolved by SDS-PAGE, transferred to nitrocellulose membranes and immunoblotted against the respective antibodies.

Supplementary References

Adams, P.D., Afonine, P.V., Bunkóczi, G., Chen, V.B., Echols, N., Headd, J.J., Hung, L.-W., Jain, S., Kapral, G.J., Grosse Kunstleve, R.W., et al. (2011). The Phenix software for automated determination of macromolecular structures. Methods *55*, 94–106.

Battye, T.G.G., Kontogiannis, L., Johnson, O., Powell, H.R., and Leslie, A.G.W. (2011). iMOSFLM: a new graphical interface for diffraction-image processing with MOSFLM. Acta Crystallogr D Biol Crystallogr *67*, 271–281.

Berrow, N.S., Alderton, D., Sainsbury, S., Nettleship, J., Assenberg, R., Rahman, N., Stuart, D.I., and Owens, R.J. (2007). A versatile ligation-independent cloning method suitable for high-throughput expression screening applications. Nucleic Acids Res *35*, e45.

Damgaard, R.B., Fiil, B.K., Speckmann, C., Yabal, M., Stadt, U.Z., Bekker-Jensen, S., Jost, P.J., Ehl, S., Mailand, N., and Gyrd-Hansen, M. (2013). Disease-causing mutations in the XIAP BIR2 domain impair NOD2-dependent immune signalling. EMBO Mol Med *5*, 1278–1295.

Damgaard, R.B., Nachbur, U., Yabal, M., Wong, W.W.-L., Fiil, B.K., Kastirr, M., Rieser, E., Rickard, J.A., Bankovacki, A., Peschel, C., et al. (2012). The Ubiquitin Ligase XIAP Recruits LUBAC for NOD2 Signaling in Inflammation and Innate Immunity. Mol Cell.

Emsley, P., Lohkamp, B., Scott, W.G., and Cowtan, K. (2010). Features and development of Coot. Acta Crystallogr D Biol Crystallogr *66*, 486–501.

Fiil, B.K., Damgaard, R.B., Wagner, S.A., Keusekotten, K., Fritsch, M., Bekker-Jensen, S., Mailand, N., Choudhary, C., Komander, D., and Gyrd-Hansen, M. (2013). OTULIN Restricts Met1-Linked Ubiquitination to Control Innate Immune Signaling. Mol Cell *50*, 818–830.

Gyrd-Hansen, M., Darding, M., Miasari, M., Santoro, M.M., Zender, L., Xue, W., Tenev, T., da Fonseca, P.C.A., Zvelebil, M., Bujnicki, J.M., et al. (2008). IAPs contain an evolutionarily conserved ubiquitin-binding domain that regulates NF-kappaB as well as cell survival and oncogenesis. Nature Publishing Group *10*, 1309–1317.

Kamiya, Y., Uekusa, Y., Sumiyoshi, A., Sasakawa, H., Hirao, T., Suzuki, T., and Kato, K. (2012). NMR characterization of the interaction between the PUB domain of peptide:N-glycanase and ubiquitin-like domain of HR23. FEBS Lett *586*, 1141–1146.

Keusekotten, K., Elliott, P.R., Glockner, L., Fiil, B.K., Damgaard, R.B., Kulathu, Y., Wauer, T., Hospenthal, M.K., Gyrd-Hansen, M., Krappmann, D., et al. (2013). OTULIN antagonizes LUBAC signaling by specifically hydrolyzing Met1-linked polyubiquitin. Cell *153*, 1312–1326.

McCoy, A.J., Grosse Kunstleve, R.W., Adams, P.D., Winn, M.D., Storoni, L.C., and Read, R.J. (2007). Phaser crystallographic software. J Appl Crystallogr *40*, 658–674.

Winn, M.D., Ballard, C.C., Cowtan, K.D., Dodson, E.J., Emsley, P., Evans, P.R., Keegan, R.M., Krissinel, E.B., Leslie, A.G.W., McCoy, A., et al. (2011). Overview of the CCP4 suite and current developments. Acta Crystallogr D Biol Crystallogr *67*, 235–242.
